# Lung Cancer Screening Decision Aid Designed for a Primary Care Setting: A Randomized Clinical Trial

**DOI:** 10.1001/jamanetworkopen.2023.30452

**Published:** 2023-08-30

**Authors:** Marilyn M. Schapira, Rebecca A. Hubbard, Jeff Whittle, Anil Vachani, Dana Kaminstein, Sumedha Chhatre, Keri L. Rodriguez, Lori A. Bastian, Jeffrey D. Kravetz, Onur Asan, Jason M. Prigge, Jessica Meline, Susan Schrand, Jennifer V. Ibarra, Deborah A. Dye, Julie B. Rieder, Jemimah O. Frempong, Liana Fraenkel

**Affiliations:** 1 Center for Health Equity Research and Promotion (CHERP), Michael J. Crescenz Veterans Affairs (VA) Medical Center, Philadelphia, Pennsylvania; 2 Department of Medicine, University of Pennsylvania Perelman School of Medicine, Philadelphia; 3 Department of Biostatistics, Epidemiology, and Informatics, University of Pennsylvania School of Medicine, Philadelphia; 4 Division of Medicine, Clement J Zablocki VA Medical Center, Milwaukee, Wisconsin; 5 Center for Advancing Population Science, Medical College of Wisconsin, Wauwatosa; 6 Department of Medicine, Michael J Crescenz VA Medical Center, Philadelphia, Pennsylvania; 7 Division of Pulmonary, Allergy and Critical Care, Department of Medicine, University of Pennsylvania Perelman School of Medicine, Philadelphia; 8 Department of Organizational Dynamics, School of Arts & Sciences, University of Pennsylvania, Philadelphia; 9 Department of Psychiatry, University of Pennsylvania, Philadelphia; 10 CHERP, VA Pittsburgh Healthcare System, Pittsburgh, Pennsylvania; 11 Department of Medicine, Yale University, New Haven, Connecticut; 12 VA Connecticut Healthcare System, West Haven; 13 The Stevens Institute of Technology, School of Systems and Enterprise, Hoboken, New Jersey; 14 Office of Research, Clement J. Zablocki VA Medical Center, Milwaukee, Wisconsin; 15 Berkshire Health Systems, Pittsfield, Massachusetts

## Abstract

**Importance:**

Guidelines recommend shared decision-making prior to initiating lung cancer screening (LCS). However, evidence is lacking on how to best implement shared decision-making in clinical practice.

**Objective:**

To evaluate the impact of an LCS Decision Tool (LCSDecTool) on the quality of decision-making and LCS uptake.

**Design, Setting, and Participants:**

This randomized clinical trial enrolled participants at Veteran Affairs Medical Centers in Philadelphia, Pennsylvania; Milwaukee, Wisconsin; and West Haven, Connecticut, from March 18, 2019, to September 29, 2021, with follow-up through July 18, 2022. Individuals aged 55 to 80 years with a smoking history of at least 30 pack-years who were current smokers or had quit within the past 15 years were eligible to participate. Individuals with LCS within 15 months were excluded. Of 1047 individuals who were sent a recruitment letter or had referred themselves, 140 were enrolled.

**Intervention:**

A web-based patient- and clinician-facing LCS decision support tool vs an attention control intervention.

**Main Outcome and Measures:**

The primary outcome was decisional conflict at 1 month. Secondary outcomes included decisional conflict immediately after intervention and 3 months after intervention, knowledge, decisional regret, and anxiety immediately after intervention and 1 and 3 months after intervention and LCS by 6 months.

**Results:**

Of 140 enrolled participants (median age, 64.0 [IQR, 61.0-69.0] years), 129 (92.1%) were men and 11 (7.9%) were women. Of 137 participants with data available, 75 (53.6%) were African American or Black and 62 (44.3%) were White; 4 participants (2.9%) also reported Hispanic or Latino ethnicity. Mean decisional conflict score at 1 month did not differ between the LCSDecTool and control groups (25.7 [95% CI, 21.4-30.1] vs 29.9 [95% CI, 25.6-34.2], respectively; *P* = .18). Mean LCS knowledge score was greater in the LCSDecTool group immediately after intervention (7.0 [95% CI, 6.3-7.7] vs 4.9 [95% CI, 4.3-5.5]; *P* < .001) and remained higher at 1 month (6.3 [95% CI, 5.7-6.8] vs 5.2 [95% CI, 4.5-5.8]; *P* = .03) and 3 months (6.2 [95% CI, 5.6-6.8] vs 5.1 [95% CI, 4.4-5.8]; *P* = .01). Uptake of LCS was greater in the LCSDecTool group at 6 months (26 of 69 [37.7%] vs 15 of 71 [21.1%]; *P* = .04).

**Conclusions and Relevance:**

In this randomized clinical trial of an LCSDecTool compared with attention control, no effect on decisional conflict occurred at 1 month. The LCSDecTool used in the primary care setting did not yield a significant difference in decisional conflict. The intervention led to greater knowledge and LCS uptake. These findings can inform future implementation strategies and research in LCS shared decision-making.

**Trial Registration:**

ClinicalTrials.gov Identifier: NCT02899754

## Key Points

**Question:**

Can a lung cancer screening (LCS) decision tool designed for use in a primary care clinical setting improve informed LCS decision-making?

**Findings:**

In this randomized clinical trial of 140 participants, no statistically significant difference in decisional conflict was found at 1 month after intervention (25.7 vs 29.9, on a scale of 0 [none] to 100 [high]). Analysis of secondary outcomes suggests that the LCS decision tool intervention improved LCS knowledge without increasing general anxiety or lung cancer worry.

**Meaning:**

This patient decision aid designed for use in a primary care setting can inform future research to advance LCS shared decision-making interventions.

## Teaser

This randomized clinical trial evaluates the efficacy of a web-based patient- and clinician-facing lung cancer screening patient decision aid compared with an attention control intervention on the quality of decision-making and uptake of lung cancer screening in a population of Veterans Affairs patients.

## Introduction

Lung cancer screening (LCS) with low-dose computed tomography decreases lung cancer mortality among older persons who smoke heavily and are healthy enough to undergo definitive therapy for early-stage lung cancer.^1,2^ The National Lung Screening Trial^1^ and the Dutch-Belgian Randomized Lung Cancer Screening Trial^2^ reported 20% and 25% reductions in lung cancer mortality in screened and control groups, respectively. National guideline committees in the US and Europe recommend LCS among those meeting eligibility criteria based on age, smoking history, and fitness for surgical treatment of lung cancer. The updated 2021 US Preventive Services Task Force (USPSTF) provided LCS with a grade B recommendation for adults aged 50 to 80 years with at least a 20 pack-year history of smoking and are current smokers or have quit within the past 15 years.^3^ The 2021 USPSTF guideline states that shared decision-making (SDM) is important when clinicians and patients discuss LCS. In addition to highlighting the benefits of LCS, this guideline notes that LCS does not prevent most lung cancer deaths and potential harms, including false-positive results, incidental findings that may lead to subsequent testing and treatment, overdiagnosis, and the risks of radiation exposure. These trade-offs underscore the need for meaningful SDM conversations between clinicians and patients prior to initiating LCS.

Analysis of the Veteran Affairs (VA) Central Cancer Registry indicates that lung cancer is the second most commonly diagnosed cancer among men and women in both VA and general US populations.^4^ The greater comorbidity burden among veterans compared with the US general population may increase the effect of harms related to LCS.^5^ For example, the higher incidence of mental health disorders could heighten the anxiety associated with false-positive test results, diagnosis of incidental findings, and the risk associated with radiation exposure. In an analysis of data through 2019,^6^ rates of LCS among eligible veterans varied by state from less than 1% to greater than 20%. Thus, the veteran population would particularly benefit from interventions to increase access to informed and value-aligned decision-making for LCS.

Patient decision aids (PDAs) for LCS in both veteran and general populations have improved decision quality in before-and-after single-group study designs.^7,8,9,10,11,12,13^ A number of SDM interventions including PDAs have also been evaluated in randomized clinical trials (RCTs).^14,15,16,17,18^ In sum, this literature supports the use of a PDA to improve knowledge and increase preparation for decision-making. However, we lack evidence of the effect of PDAs on LCS uptake. Moreover, to the best of our knowledge, PDAs have not been studied using RCTs in the context of primary care settings, where screening decisions are usually made.

The objective of this study was to evaluate the efficacy of a web-based patient- and clinician-facing LCS PDA compared with an attention control intervention on the quality of decision-making and uptake of LCS. We developed an LCS decision tool (LCSDecTool) for use by veterans eligible for LCS and enrolled in primary care at a VA Medical Center.^19^ We hypothesized that use of the LCSDecTool would decrease decisional conflict at 1 month. As a secondary outcome, we hypothesized that there would be a decrease in uptake of LCS in the LCSDecTool compared with the control intervention due to increased awareness of harms associated with LCS. Additional secondary outcomes were LCS knowledge, decisional regret, anxiety, and lung cancer worry.

## Methods

This RCT was reviewed and approved by the institutional review boards of all participating sites, and all participants provided written or oral informed consent. The study protocol is included in Supplement 1. We followed the Consolidated Standards of Reporting Trials (CONSORT) reporting guideline.

We conducted an RCT using a parallel design with a 1:1 allocation ratio. Recruitment sites included VA Medical Centers in Philadelphia, Pennsylvania, West Haven, Connecticut, and Milwaukee, Wisconsin. We used the National VA Corporate Data Warehouse to identify veterans potentially eligible for LCS who had an upcoming appointment within 3 weeks. We used prescription data (varenicline tartrate or nicotine replacement therapy) or positive screen results for active smoking in primary care or dental clinic notes to identify potential participants. We then performed a focused electronic record review and telephone screening to further assess eligibility. Inclusion criteria were (1) age 55 to 80 years, (2) active smokers or those who quit smoking within the past 15 years, (3) history of at least 30 pack-years of smoking, and (4) an upcoming appointment in primary care within 3 weeks. Exclusion criteria were (1) a cancer diagnosis, except for nonmelanoma skin cancer or prostate cancer not requiring active treatment, and (2) a primary care clinician assessment of life expectancy less than 2 years.

Participant race and ethnicity data were collected via self-report. Participants were given the option of identifying as 1 or more of the following categories: African American or Black, American Indian or Alaska Native, Asian, Hispanic or Latino, Native Hawaiian or Other Pacific Islander, and White. We collected these data to evaluate the generalizability of our findings and to conduct prespecified exploratory analyses among participants who identified as African American or Black.

### Description of Intervention

We developed the LCSDecTool with a user-centered design as guided by International Patient Decision Aid Standards.^20^ The LCSDecTool was designed to be used independently by the patient before the clinic visit with the option to share some components with the clinician during the clinic visit. The features include an overview of LCS using a simulated patient-clinician dialogue, interactive knowledge boxes providing brief information on 6 key attributes related to LCS, a pictograph representing LCS outcomes, a value elicitation exercise, smoking cessation advice, mental health resources, and the option to request a referral to a smoking cessation clinic or to a behavioral health clinician to support smoking cessation efforts. The tool includes a text field where a patient can enter questions for their clinician. A summary page is created by the LCSDecTool that displays participant value ratings and questions entered for the clinician. Users can advance through the tool at their own pace. From the opening page, a clinician can access a clinician portal that directly connects to the pictograph of LCS outcomes and the summary page. A link to the LCSDecTool is provided in eMethods in Supplement 2.

### Description of the Control Intervention

The web-based attention control intervention was entitled Cancer Prevention: An Information Guide. The 10-page guide provides general information on cancer prevention and the USPSTF screening guidelines for breast, colon, cervical, and lung cancer. Users could advance through the program at their own pace. A link to the control intervention is provided in eMethods in Supplement 2.

### Study Protocol

Participants had a study visit on the day of or the day prior to their scheduled primary care appointment. A consent process including a participant signature was completed for those with in-person research visits and an oral consent process for persons who had a remote study visit. Following informed consent, the participant logged on to the LCSDecTool or control program. Upon completion, those randomized to the LCSDecTool had the option to print or save the summary page and to bring the LCSDecTool to the clinic visit. Modifications made to the protocol during the pandemic included enabling use during telephone and virtual video visits.^21^

### Baseline Measures

Baseline measures included health literacy assessed by the Rapid Estimate of Adult Literacy in Medicine,^22^ which asks respondents to read and pronounce 66 medically related words, is scored from 0 to 66 (higher scores indicate greater health literacy) and categorizes persons in terms of grade level reading skills. Health numeracy was assessed with the Numeracy Understanding in Medicine Instrument Short Form, an 8-item scale with scores ranging from 0 (low numeracy) to 8 (high numeracy).^23^ We also measured LCS knowledge on a 12-item validated measure (including items on lung cancer mortality reduction, false-positive results, overdiagnosis, incidental findings, and radiation exposure), sociodemographic factors, comorbidities, state anxiety using the State-Trait Anxiety Inventory, and quality of life as assessed by the 36-Item Short Form Health Survey.^24,25^

### Outcome Measures

The primary outcome was decisional conflict as measured by the Decisional Conflict Scale (DCS) at 1 month after intervention. The DCS is a 16-item scale with 5 subscales: Informed, Values Clarity, Support, Uncertainty, and Effective Decision. Scores range from 0 (low decisional conflict) to 100 (high decisional conflict). Scores on the DCS lower than 25 are associated with implementing decisions and greater than 37.5 with decision delay or feeling unsure about implementation.^26^ Secondary outcomes included decisional conflict immediately after intervention and 3 months after intervention, LCS knowledge, decisional regret,^27^ and anxiety immediately after intervention and 1 and 3 months after intervention and LCS by 6 months. Outcomes were obtained immediately after the clinic visit and at 1 and 3 months. Patient-reported SDM was assessed by the CollaboRATE 3-item scale with scores ranging from 1 (low SDM) to 4 (high SDM).^28,29^ Outcomes were assessed by surveys administered in person or by telephone. Uptake of LCS was assessed at 6 and 9 months by electronic record review.

### Randomization Sequence Generation

We used a random sequence generator programmed into the web-based tool to assign each participant upon login to the LCSDecTool vs control intervention. Randomization was blocked by study site. The random allocation sequence was generated by the website developers and study staff. The research associates who enrolled participants (J.M.P., J.M., J.V.I., D.A.D., and J.B.R.) were not aware of group assignment prior to the enrolled participant logging on to the web-based program.

### Statistical Analysis

A sample size of 148 participants provided 80% power to detect a medium effect size of 0.49 on the DCS with α = .05, assuming 10% loss to follow-up and an SD of 11.9,^30^ for an analytic sample of 134. We described baseline characteristics using counts and percentages or median (IQR) for dichotomous and continuous variables, respectively. We report outcomes at baseline, immediately after the intervention, and at 1 and 3 months in the total cohort and stratified by study groups. We calculated unadjusted means and 95% CIs for primary and exploratory outcomes at each time point. We also calculated differences in means between groups and their 95% CIs. We calculated *P* values using a linear mixed-effects model adjusted for study site and including random subject intercepts. A prespecified exploratory analysis was conducted among participants who identified as African American or Black for the primary outcome and LCS uptake at 6 months (a secondary outcome) to explore heterogeneity in outcomes across race groups. In a sensitivity analysis, we estimated LCS uptake within 9 months. Differences in the probability of receiving LCS and their 95% CI and *P* values were adjusted for study site using logistic regression followed by marginal standardization. Finally, unadjusted means were compared between the LCSDecTool and control groups for CollaboRATE scores. We considered a 2-sided *P* < .05 to be statistically significant for our primary outcome. All statistical analyses were conducted using R, version 4.2.0 (R Project for Statistical Computing).

## Results

### Study Cohort

We identified 6162 potential participants from the Corporate Data Warehouse VA Database, with 1043 meeting inclusion criteria after electronic record review and mailed recruitment letters. Four participants were self-referred. Among these, 140 persons were successfully contacted, were eligible, and agreed to participate. Sixty-nine participants were randomized to the experimental intervention and 71 to the control group (Figure). Of the 140 participants, 129 (92.1%) were men, and 11 (7.9%) were women. Of 137 participants with data available, 75 (53.6%) were African American or Black and 62 (44.3%) were White; 4 participants (2.9%) also reported Hispanic or Latino ethnicity. The median age was 64.0 (IQR, 61.0-69.0) years. The median duration of smoking was 43.4 (IQR, 37.5-52.0) pack-years. Additional demographic, smoking history, and baseline clinical characteristics of the cohort are in Table 1.

**Figure.**
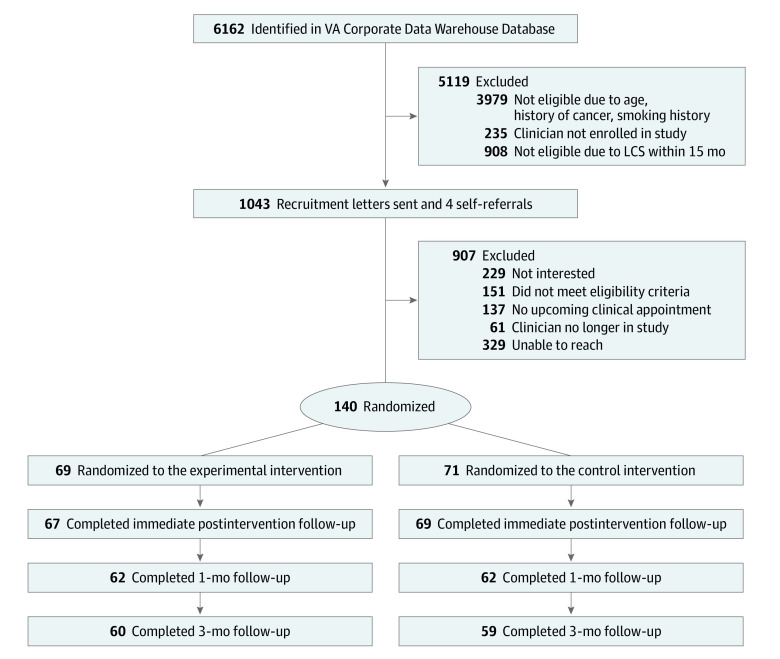
Study Flow Diagram LCS indicates lung cancer screening; VA, Veterans Affairs.

**Table 1.** Baseline Participant Characteristics

Characteristic	Participant group^a^		
	Total (N = 140)	Experimental intervention (n = 69)	Control (n = 71)
Age, median (IQR), y	64.0 (61.0-69.0)	64.0 (61.0-69.0)	64.0 (61.8-69.5)
Sex			
Men	129 (92.1)	61 (88.4)	68 (95.8)
Women	11 (7.9)	8 (11.6)	3 (4.2)
Race			
African American or Black	75 (53.6)	42 (60.9)	33 (46.5)
White	62 (44.3)	26 (37.7)	36 (50.7)
Missing	3 (2.1)	1 (1.4)	2 (2.8)
Hispanic or Latino ethnicity	4 (2.9)	1 (1.4)	3 (4.2)
Educational level			
Grade school	4 (2.9)	3 (4.4)	1 (1.4)
High school or GED	52 (37.1)	28 (40.6)	24 (33.8)
Some college or university	72 (51.4)	33 (47.8)	39 (54.9)
≥4 y College or university	9 (6.4)	3 (4.4)	6 (8.4)
After baccalaureate	3 (2.1)	2 (2.9)	1 (1.4)
Income, $			
0-25 000	43 (30.7)	28 (40.6)	15 (21.1)
>25 000-50 000	56 (40.0)	24 (34.8)	32 (45.1)
>50 000-75 000	20 (14.3)	11 (15.9)	9 (12.7)
>75 000-100 000	4 (2.9)	1 (1.4)	3 (4.2)
>100 000	5 (3.6)	2 (2.9)	3 (4.2)
Prefer not to say	12 (8.6)	3 (4.4)	9 (12.7)
Duration of smoking, median (IQR), pack-years	43.4 (37.5-52.0)	40.5 (35.0-50.0)	45.0 (39.0-54.0)
Current smoker	92 (65.7)	51 (73.9)	41 (57.7)
PTSD	62 (44.3)	31 (44.9)	31 (43.7)
Depression	61 (43.6)	33 (47.8)	28 (39.4)
Anxiety	47 (33.6)	24 (34.8)	23 (32.4)
Arthritis	65 (46.4)	30 (43.5)	35 (49.3)
Hypertension	49 (35.0)	25 (36.2)	24 (33.8)
Diabetes	41 (29.3)	18 (26.1)	23 (32.4)
Emphysema (COPD)	28 (20.0)	14 (20.3)	14 (19.7)
Heart disease	26 (18.6)	9 (13.0)	17 (23.9)
Asthma	7 (5.0)	4 (5.8)	3 (4.2)
Health literacy REALM score (grade level)^b^			
0-18 (≤3rd)	1 (0.7)	1 (1.4)	0
19-44 (4th-5th)	2 (1.4)	0	2 (2.8)
45-60 (6th-7th)	8 (5.7)	3 (4.4)	5 (7.0)
61-66 (8th-9th)	129 (92.1)	65 (94.2)	64 (90.1)
s-NUMi score, median (IQR)^c^	5.0 (4.0-5.0)	4.0 (4.0-5.0)	5.0 (4.0-5.0)
SF-36 score, median (IQR)^d^			
General health	55.0 (40.0-68.8)	55.0 (40.0-65.0)	55.0 (40.0-70.0)
Emotional well-being	68.0 (53.0-80.0)	64.0 (55.0-76.0)	70.0 (53.0-84.0)
Social functioning	62.5 (50.0-87.5)	62.5 (40.6-75.0)	62.5 (50.0-87.5)
Limitations due to physical health	50.0 (0.0-100.0)	37.5 (0.0-100.0)	50.0 (0.0-100.0)
Limitations due to emotional health	66.7 (0.0-100.0)	33.3 (0.0-100.0)	100.0 (0.0- 100.0)
Energy or fatigue	50.0 (40.0-60.0)	50.0 (40.0-60.0)	50.0 (42.5-60.0)
Pain	45.0 (22.5-67.5)	45.0 (22.5-67.5)	45.0 (32.5-67.5)
State-Trait Anxiety Inventory, median (IQR)^e^	37.0 (28.2-46.0)	39.0 (33.0-46.0)	35.0 (26.0-46.0)
Lung cancer worry score, median (IQR)^f^	6.0 (4.0-7.0)	6.0 (4.0-7.0)	6.0 (4.0-7.0)

Abbreviations: COPD, chronic obstructive pulmonary disease; GED, General Educational Development; SF-36, 36-item Short Form Health Survey; s-NUMi, Numeracy Understanding in Medicine Instrument Short Form; PTSD, posttraumatic stress disorder; REALM, Rapid Estimate of Adult Literacy in Medicine.

^a^ Unless otherwise indicated, data are expressed as No. (%) of participants. Percentages are rounded and therefore may not total 100.

^b^ Scores range from 0 (low health literacy) to 66 (high health literacy).

^c^ Scores range from 0 (low numeracy) to 8 (high numeracy). Includes 131 participants (total), 65 in the experimental intervention group, and 66 in the control group.

^d^ Scores range from 0 (low quality of life) to 100 (high quality of life).

^e^ Scores range from 20 (low anxiety) to 80 (high anxiety).

^f^ Scores range from 3 (low worry) to 13 (high worry).

### Primary Outcome

Of the 69 participants randomized to the experimental intervention, 68 (98.6%) logged into the tool and completed the tool as indicated by a generated summary page. The unadjusted primary outcome of mean DCS score at 1 month after intervention for the LCSDecTool and control groups was 25.7 (95% CI, 21.4-30.1) and 29.9 (95% CI, 25.6-34.2), respectively. The mean difference in the LCSDecTool and control scores at 1 month was −4.1 (adjusted 95% CI, −9.9 to 1.8; *P* = .18).

### Secondary Outcomes

The mean unadjusted outcome of DCS scores at the immediate postintervention assessment for the LCSDecTool and control groups were 22.2 (95% CI, 18.3-26.0) and 31.1 (95% CI, 26.1-36.0), respectively. The mean difference at the immediate postintervention time point was −8.2 (adjusted 95% CI, −13.8 to −2.5; *P* = .004) (Table 2 and eFigure in Supplement 2). The mean unadjusted outcomes of the DCS subscale Informed at the immediate postintervention assessment for the LCSDecTool and control groups were 24.3 (95% CI, 19.9-28.6) and 39.3 (95% CI, 33.5-45.1), respectively, with a mean difference of −14.6 (adjusted 95% CI, −21.7 to −7.5; *P* < .001) (Table 2). The mean unadjusted outcomes of the DCS subscale Effective Decision at the immediate postintervention assessment for the LCSDecTool and control groups were 19.5 (95% CI, 15.2-23.8) and 26.7 (95% CI, 21.4-32.0), respectively, with a mean difference of −6.9 (adjusted 95% CI, −12.0 to −0.9; *P* = .02) (Table 2). The exploratory findings among participants who identified as African American or Black did not differ from the primary findings at 1 month after intervention (eTable in Supplement 2).

**Table 2.** Study Outcomes by Follow-Up Time Point^a^

Outcome	Immediately after intervention				1 mo After intervention				3 mo After intervention			
	Group mean (95% CI)		Between-group difference (95% CI)	*P* value	Group mean (95% CI)		Between-group difference (95% CI)	*P* value	Group mean (95% CI)		Between-group difference (95% CI)	*P* value
	Experimental intervention	Control			Experimental intervention	Control			Experimental intervention	Control		
DCS total scale	22.2 (18.3 to 26.0)	31.1 (26.1 to 36.0)	−8.2 (−13.8 to −2.5)	.004	25.7 (21.4 to 30.1)	29.9 (25.6 to 34.2)	−4.1 (−9.9 to 1.8)	.18	24.2 (20.8 to 27.6)	27.5 (23.3 to 31.7)	−2.9 (−8.9 to 3.0)	.33
DCS Uncertainty subscale	22.9 (18.3 to 27.4)	30.1 (24.7 to 35.6)	−6.6 (−13.4 to 0.2)	.06	25.0 (20.2 to 29.8)	31.5 (25.8 to 37.1)	−5.8 (−12.9 to 1.3)	.11	26.1 (21.5 to 30.7)	29.3 (23.6 to 35.0)	−2.3 (−9.6 to 5.0)	.53
DCS Informed subscale	24.3 (19.9 to 28.6)	39.3 (33.5 to 45.1)	−14.6 (−21.7 to 7.5)	<.001	26.6 (21.3 to 32.0)	35.1 (29.7 to 40.4)	−8.3 (−15.7 to −0.9)	.03	27.7 (22.0 to 33.3)	31.5 (25.6 to 37.5)	3.4 (−11.0 to 4.2)	.38
DCS Values Clarity subscale	24.0 (19.2 to 28.8)	34.1 (28.7 to 39.5)	−9.5 (−16.2 to −2.8)	.01	31.5 (26.4 to 36.5)	35.5 (30.2 to 40.8)	−3.9 (−10.9 to 3.1)	.28	26.1 (21.9 to 30.3)	31.1 (25.8 to 36.4)	−4.9 (−12.1 to 2.3)	.18
DCS Support subscale	21.6 (17.2 to 25.9)	26.1 (21.0 to 31.2)	−4.1 (−10.4 to 2.1)	.19	23.2 (18.4 to 27.9)	24.6 (20.1 to 29.1)	−1.3 (−7.8 to 5.2)	.70	23.2 (18.6 to 27.8)	22.8 (18.3 to 27.2)	0.4 (−6.2 to 7.1)	.89
DCS ED subscale	19.5 (15.3 to 23.8)	26.7 (21.4 to 32.0	−6.9 (−12.8 to −0.9)	.02	20.2 (15.9 to 24.6)	23.5 (18.8 to 28.2)	−3.0 (−9.3 to 3.2)	.34	19.9 (16.6 to 23.2)	24.4 (20.0 to 28.8)	−3.9 (−10.2 to 2.5)	.23
LCS knowledge	7.0 (6.3 to 7.7)	4.9 (4.3 to 5.5)	2.0 (1.2 to 2.8)	<.001	6.3 (5.7 to 6.8)	5.2 (4.5 to 5.8)	0.9 (0.1 to 1.8)	.03	6.2 (5.6 to 6.8)	5.1 (4.4 to 5.8)	1.1 (0.3 to 2.0)	.01
Decisional regret	32.6 (30.1 to 35.1)	34.5 (31.9 to 37.1)	−1.9 (−5.2 to 1.5)	.27	32.2 (29.6 to 34.8)	34.7 (32.2 to 37.2)	−2.8 (−6.3 to 0.7)	.12	32.5 (30.1 to 35.0)	34.3 (31.9 to 36.7)	−2.0 (−5.6 to 1.5)	.26
Anxiety STAI	33.5 (30.7 to 36.3)	32.8 (29.8 to 35.9)	0.4 (−3.9 to 4.6)	.86	37.2 (34.0 to 40.4)	35.2 (31.3 to 39.0)	2.3 (−2.0 to 6.6)	.30	36.6 (33.6 to 39.7)	38.2 (33.9 to 42.4)	−0.7 (−5.1 to 3.6)	.74
Lung cancer worry	5.9 (5.2 to 6.5)	6.4 (5.9 to 6.9)	−0.5 (−1.3 to 0.2)	.18	6.3 (5.7 to 6.8)	6.3 (5.7 to 7.0)	−0.2 (−1.0 to 0.6)	.58	5.4 (4.9 to 6.0)	5.9 (5.3 to 6.6)	−0.6 (−1.4 to 0.2)	.16

Abbreviations: DCS, Decisional Conflict Scale; ED, Effective Decision; LCS, lung cancer screening; STAI, State Trait Anxiety Index.

^a^ Within-group means and 95% CIs are unadjusted. Between-group differences in means and their 95% CIs as well as *P* values are based on linear mixed-effects model adjusted for baseline value and site and including random participant intercepts.

The mean scores for LCS knowledge were greater in the LCSDecTool vs control groups immediately after intervention (7.0 [95% CI, 6.3-7.7] vs 4.9 [95% CI, 4.3-5.5]; mean difference, 2.0 [95% CI, 1.2-2.8]; *P* < .001) and remained greater at 1 month (6.3 [95% CI, 5.7-6.8] vs 5.2 [95% CI, 4.5-5.8]; mean difference, 0.9 [95% CI, 0.1-1.8]; *P* = .03) and 3 months (6.2 [95% CI, 5.6-6.8] vs 5.1 [95% CI, 4.4-5.8]; mean difference, 1.1 [95% CI, 0.3-2.0]; *P* = .01) (Table 2 and eFigure in Supplement 2. There were no significant differences in decisional regret, state anxiety, or lung cancer worry scores between the LCSDecTool and control groups overall (Table 2 and eFigure in Supplement 2) or in the subgroup analysis at the 1-month time point among participants who identified as African American or Black (eTable in Supplement 2).

The mean CollaboRATE scores immediately after intervention were 2.9 (95% CI, 2.6-3.1) and 2.6 (95% CI, 2.3-2.9; *P* = .15) for the LCSDecTool vs control groups, respectively. Among those randomized to the LCSDecTool, 23 (33.3%) clicked a radio button to indicate they would like to speak with a mental health clinician to help with smoking cessation and 26 (37.7%) indicated they would like to speak with their primary care clinician about smoking cessation. The mean (SD) time to complete the CollaboRATE was 1.4 (6.0) minutes for the LCSDecTool group compared with 5.2 (2.5) minutes for the control group.

### LCS Uptake

Among study participants at 6 months after intervention, the proportion receiving an LCS scan was greater in the LCSDecTool (26 of 69 [37.7%]) vs the control (15 of 71 [21.1%]) groups. The adjusted difference between proportions was statistically significant (16.0% [95% CI, 1.8%-30.2%]; *P* = .04). The higher LCS uptake in the LCSDecTool group persisted at 9 months (31 of 69 [44.9%] vs 18 of 71 [25.4%]; *P* = .02) (Table 3). In an exploratory subgroup analysis among participants who identified as African American or Black, uptake of LCS at 6 months in the LCSDecTool group was 15 (35.7%) compared with 7 (21.2%) in the control group (*P* = .17).

**Table 3.** Lung Cancer Screening Uptake^a^

LCS scan uptake, mo	Participant group, No (%)		Difference (95% CI)	*P* value
	Experimental intervention	Control		
6	26 (37.7)	15 (21.1)	16.0 (1.8-30.2)	.04
9	31 (44.9)	18 (25.4)	18.8 (4.4-33.2)	.02

Abbreviation: LCS, lung cancer screening.

^a^ Confirmed by electronic medical record review at 6 and 9 months following the index clinical visit.

## Discussion

In this RCT of an LCS PDA, contrary to our hypothesis, we found no statistically significant difference in our primary outcome of decisional conflict between the LCSDecTool and control groups at 1 month after intervention. However, LCS knowledge increased immediately after intervention and remained higher over time, and LCS uptake was higher in the LCSDecTool group (37.7% vs 21.1%). There was no difference in decisional regret or general anxiety between groups.

The trajectory of decisional conflict in our study indicates a lower level of decisional conflict in the LCSDecTool group vs the control group immediately after intervention, with differences mitigated at 1 and 3 months. Decisional conflict may be most subject to change immediately following use of a PDA due to the salience of new information and value clarification experience. However, some users may continue to process information and weigh risks and benefits over a period of time; measurement of decisional conflict at 1 month may reflect the results of this process.

The persistent increase in LCS knowledge among the LCSDecTool group vs the control group indicates effective communication of concepts relevant to LCS, including the potential benefits and harms. The higher uptake of LCS among the LCSDecTool group could reflect increased awareness of LCS, greater knowledge about LCS benefits and harms, and an individual value assessment that the benefits outweighed the harms. Participants also had the opportunity to discuss LCS with clinicians, as the intervention was timed to coincide with a scheduled clinic visit. Although both interventions provided participants with LCS guidelines, the LCSDecTool, which was designed to facilitate an informed and value-aligned decision process, may have worked through those mechanisms.

Shared decision-making for LCS is widely recommended by professional organizations, guideline committees, and payers.^31,32^ However, implementation studies of SDM for LCS have met with mixed results.^33,34^ Volk et al^18^ evaluated an implementation strategy of a video-delivered PDA entitled *Lung Cancer Screening: Is It Right for Me?* The PDA was compared with a standard educational intervention among persons calling a smoking cessation hotline. Those randomized to the PDA compared with the control group had lower levels of decisional conflict, as indicated by 2 DCS subscales (Informed and Values Clarity) and increased LCS knowledge. However, there was no difference in LCS uptake between groups.^18^ Our study protocol differed in delivering the LCSDecTool prior to a primary care visit, providing an opportunity for discussion and clinical decision-making regarding LCS.

In a cluster randomized trial among 8 VA Medical Centers, Lowery et al^9^ compared implementation strategies (quality improvement training and academic detailing) for an LCS provider-facing, web-based decision support tool called DecisionPrecision. The tool guides clinicians to tailor an LCS SDM discussion based on individual lung cancer risk profiles. Neither implementation strategy increased the use of the tool, with clinician time constraints and competing priorities emerging as barriers.^9^

Our study has clinical and research implications. Our findings support the use of a PDA in the setting of a primary care clinic visit when a discussion can occur and a decision about LCS can be made. In addition, the secondary findings of increased uptake of LCS combined with a sustained increase in knowledge about LCS benefits and harms support the value of the LCSDecTool. Further studies are needed to elucidate the trajectory of decision quality outcomes and LCS uptake over time following the use of a PDA.

### Limitations

This study has several limitations. First, the study was powered for the primary outcome of decisional conflict at 1 month after intervention, and other findings are considered exploratory. Further, decisional conflict scores were relatively low in both groups, limiting the ability to detect clinically significant differences. Second, study participants were limited to US veterans receiving primary care in a VA Medical Center, a population that is primarily male and specific to the US, limiting generalizability. However, participants were diverse in many respects, including race, educational background, and health numeracy. Third, individuals willing to participate in a clinical trial about cancer screening may be more health conscious and inclined to participate in cancer screening, leading to selection bias. Finally, the study period included the COVID-19 pandemic. Protocol adaptations such as inclusion of remote study and clinic visits were required.^19,20,21^ However, the LCSDecTool, developed for use across computer, tablet, and smartphone platforms, was successfully applied across these platforms during remote study visits.

## Conclusions

In this randomized clinical trial of an LCSDecTool compared with an attention control intervention, we found no significant difference in the primary outcome of decisional conflict at 1 month. Secondary outcomes included a sustained increase in LCS knowledge, no difference in anxiety or lung cancer worry, and increased LCS uptake in the LCSDecTool group compared with the control group. These findings can inform future implementation strategies and research on SDM for LCS.

## Notes

Supplement 1.
Trial Protocol

Supplement 2. **eMethods.** Study Interventions **eFigure.** Quality of Decision-Making Outcomes for Knowledge and Decisional Conflict **eTable.** Analysis of Decision Quality Outcomes Among African American or Black Participants

Supplement 3.
Data Sharing Statement
