# Supplementary material for: Lung Cancer Screening Decision Aid Designed for a Primary Care Setting: A Randomized Clinical Trial
Source: JAMA Netw Open. 2023 Aug 30;6(8):e2330452. doi: 10.1001/jamanetworkopen.2023.30452 (PMC10469267; doi:10.1001/jamanetworkopen.2023.30452)
Supplement: Supplement 1. — Trial Protocol [file jamanetwopen-e2330452-s001.pdf]

CMCVAMC SPECIFIC PROTOCOL SUMMARY
Corporal Michael J. Crescenz Department of Veterans Affairs Medical Center (CMCVAMC)
Institutional Review Board (IRB)

Instructions: Click in box and start typing or click 'choose an item' and choose the applicable entry.
NOTE: If you are using a MAC, you may have difficulty with checkboxes and "choose an item" boxes.

Section 1. General Information

Protocol Title: *Incorporating Veterans' Preferences into Lung Cancer Screening Decisions- Phase 3*

CMCVAMC Protocol Version Number and Date: *Version #8 03/03/2021*

Principal Investigator (PI) Name: *Marilyn M. Schapira*

PI's Academic Degree(s): *MD, MPH*

Is the study funded? *YES* **If "yes", specify funding agency:** *VA Health Services Research & Development Service (HSR&D)*

Is a grant application requesting funds for the study currently being reviewed? *NO*

CMCVAMC is the only institution involved: *NO*

CMCVAMC is the coordinating center in which the PI is the lead investigator: *YES* **If this answer is yes, complete the next two sections:**

- **List the name(s) of the other site(s) involved**. *West Haven VA Medical Center (WHVAMC) Milwaukee VA Medical Center*
- **Provide the FederalWide Assurance (FWA) numbers for each site**. *WHVAMC 00001286, Milwaukee VAMC 00000289*

State name of coordinating center if this is not CMCVAMC. *n/a*

Describe PI's qualifications to conduct this project and attach a copy of PI's VA or NIH biosketch. Be specific in regard to PI's research experience. *NOTE: If PI does not have any prior research experience, indicate what provisions are being made to provide oversight or mentoring.* *Dr. Schapira is a Professor at the University of Pennsylvania and the Center for Health Equity Research Program (CHERP) at the Corporal Michael J. Crescenz VA Medical Center (CMVAMC) and co-Director of the Cancer Control Research Program the Abramson Cancer Center at the University of Pennsylvania (Penn). Her research has focused on risk communication and medical decision-making. Her work has advanced methods of shared decision-making including the measurement of health numeracy, communication of risk and uncertainty, and preference assessment. The clinical context of her research has been oncology, encompassing both screening and treatment decisions. Her work has been trans-disciplinary, reflecting the discipline of decision science. She collaborates with experts in clinical medicine, cognitive and social psychology, economics, education, and biostatistics. In work supported by the NCI and the American Cancer Society, she has developed a theoretically based and cross-culturally equivalent measure of health numeracy called the Numeracy Understanding in Medicine Instrument (NUMi). The NUMi is being translated into Spanish and a Computer Adaptive Version (CAT) version has been developed. She is now conducting a cohort study to evaluate the NUMi as a screening test for health numeracy that can be used in the clinical setting and communication strategies in the cancer treatment consultation. Her work has also focused on evaluating cancer screening paradigms from a population perspective. Her focus in the decision sciences is reflected in my professional leadership positions including past President of the Society for Medical Decision-making (SMDM) and*

APPROVED by CMCVAMC IRB on 03/28/2021

Associate Editor of Medical Decision-making. She assembled a trans-disciplinary team (clinical medicine, economics, biostatistics, human factors engineering, and implementation science) to develop an innovative decision support tool for Veterans who are eligible for and considering lung cancer screening. This project builds on her prior work in preference assessment and shared decision-making in the context of cancer care.

Does any research staff member have an actual and/or perceived conflict of interest with this study? *NO* If yes, explain.

Is this study a clinical trial? *YES* **If yes, specify the type.** *Phase 3*

NOTE: Only 'applicable clinical trials' must be registered on ClinicalTrials.gov. The term 'applicable clinical trial' means an applicable drug clinical trial or an applicable device clinical trial. Investigators might consider registering all other clinical trials on ClinicalTrials.gov, if journals in which they publish studies require such registration prior to starting a trial.

State the estimated length of time to complete enrollment of subjects. *24 Months*

State the expected duration of participation by individual subjects (including any follow-up, e.g., need to re-contact subject for follow-up questions prior to closure of the study). *One Study Visit; One 1-Month Phone Follow-Up and One 3-Month Phone Follow-Up*

Specify the projected date of completion of the study. *Phase 3 – March 2021*

Section 2: Participating Site Specifications

2.1. **Where will the research project be conducted? (Check all that apply)**

- ☐ **VA Inpatient Setting**
- ☑ **VA Outpatient Clinic/Office**
- ☐ **VA Laboratories**
- ☐ **Subject Homes**
- ☐ **University of Pennsylvania**
- ☑ **Community Based Outpatient Clinics (CBOCs)** (Gloucester, NJ, Saracini (Horsham, PA), Camden, NJ, Burlington, NJ, Fourth Street VA Clinic, Chestnut Street VA Clinic, West Philadelphia VA Clinic)
- ☑ **Other *(Specify)*:** *WHVAMC*

2.2. **If research is conducted at a non-VA site, please specify where and how much of the project will be conducted at that location**.

Dr. Schapira has an office at Penn and some of the work with coded data may occur at that office through a VPN connection to VA Secure Computers. The office number 1316 Blockley and the location is 423 Guardian Drive, Philadelphia, PA, 19104. The office is located in close proximity to the CMCVAMC. All interactions with VA subjects occur at the VA.

Section 3: Introduction

3.1. **Provide scientific background and rationale for study. Including summary of gaps in current knowledge, relevant data, and how the study will add to existing knowledge.**

Lung cancer is a significant health issue, and especially so within the Veteran population. Lung cancer is the top cause of cancer-related deaths, and this risk is higher for Veterans because of age and smoking history [1]. The majority of lung cancer prevention has focused on ending smoking, but a recent study that compared low dose computerized tomography scans (LDCT) to simple chest-x-ray scans saw a 20% decrease in cancer specific mortality rates [2]. Following additional evidence [3], the US Preventive Services Task Force and American Cancer Society, along with a number of other cancer

APPROVED by CMCVAMC IRB on 03/28/2021

preventative organizations, issued guidelines in favor of annual lung cancer screenings for older people with a history of smoking [4-6]. However, there are concerns that with more screenings an increase in false positives and over diagnosis will occur. The effectiveness of lung cancer screening may also vary by gender, and initial data suggests some Veterans are reluctant to enter an intensive screening process [7]. As the Department of Veteran Affairs expands the paradigm of lung cancer control to include lung cancer screening, it is critical to develop and test decision support tools that 1) educate Veterans regarding expected outcomes, 2) incorporate Veteran preferences and values into the decision-making process, and 3) encourage shared decision-making between Veterans and providers

The VA method of lung cancer screening is unique in that it is designed to increase assessment rates and support among Veterans. Lung cancer screening criteria includes age and the modifiable behavior of smoking. As stated before, the high rate of false positives negatively impacts screening participation, so the assessment of patient preferences will inform participants of strategies to encourage adherence to the program [8]. Comorbidity must also be taken into account, as the target screening population is older [9], and at a higher risk for vascular diseases than younger people. A successful paradigm will include these concerns. The unique aspects of the lung cancer screening paradigm increase the importance of preference assessment as part of an informed decision-making process.

A recent Cochrane meta-analysis found that decision aids such as conjoint analysis and best-worse scaling (commonly called discrete choice experiments) can improve receptiveness to lung cancer screening methods [10]. However, these tools have not been widely used in clinical practice, and these assessment methods would have to be geared towards Veteran population and require systems to integrate decision support with clinical practice. This proposal will address these gaps by evaluating the usability and acceptability of an explicit preference assessment method as part of a lung cancer screening decision support tool (LCSDecTool) designed for Veterans.

Significance

Veterans are at higher risk of lung cancer due to their increased smoking rate when compared to the general population, both when active and following service time [1]. Eight VA Medical Centers have recently completed studies that demonstrate the need for effective screening tactics. The PI of this proposal (Schapira) has completed pilot work with HSR&D Research in this area. and the demonstration projects across 8 sites in the VA health care system found that 56% of the Veterans offered screening agreed to the tests. Before expanding further, it is imperative that decision-support tools are present to encourage participation in lung cancer screening programs. By developing these strategies now, patient preferences can be accounted for in the study guidelines. (Figure 1).

Figure 1: Lung Cancer Screening Evidence Generation and Translation

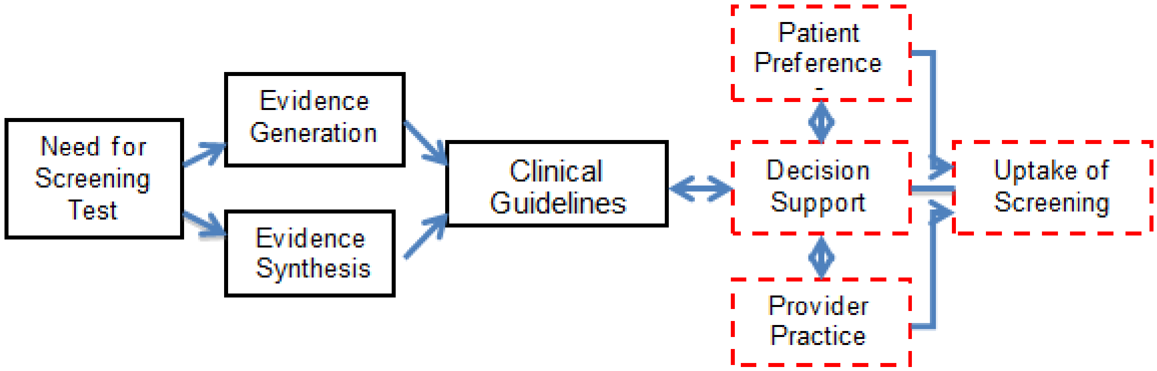

This research is based on the theoretical frameworks of shared decision-making (SDM), implementation science, and technology adoption. SDM incorporates normative and dual process reasoning theories. Dual process theory recognizes the role of systematic reasoning, which is thought to lead to decisions that people are more likely to follow through with [11]. Implementing a decision support tool that

APPROVED by CMCVAMC IRB on 03/28/2021

provides evidence-based information clearly reduces the barriers to dual process reasoning leading to higher decision quality and more accurate risk perception [12-15].
This study simultaneously collects data to gauge effectiveness of intervention and implementation. Data collection pertaining to process evaluation will be guided by the Promoting Action on Research Implementation in Health Services (PARIHS) implementation framework [16-18] and the Technology Acceptance Model (TAM) [19]. This team is qualified to conduct this work, as the core and coinvestigators have a number of relevant experiences to draw from regarding this study.

Pilot data to support this program was conducted at Penn in two parts: 1) an HSR&D pilot (PI Schapira) of preference assessment and risk communication among Veterans considering lung cancer screening, and 2) a Center for the Evaluation of Patient Aligned Care Teams (CE-PACT) pilot grant to develop a web-based health numeracy tutorial for patients enrolled in a Patient Aligned Care Team (PACT) at the CMCVAMC. Qualitative interviews with Veterans were conducted in the HSR&D pilot to define the most critical attributes of lung cancer screening. In the pilot, it was seen that Veterans found pictographs to be useful for conveying expected outcomes, along with gradations indicating risk estimates, and a "margin of error" interval analogy. Veteran concerns included the financial and emotional strain of follow-up tests resulting from false positives. Fifty-one percent (51%) of study participants were African American, 86% were current smokers, and 47% had at least 30 pack years of smoking (this means 1 pack per day for 30 years or 2 packs a day for 15 years). Participants were asked to choose the attributes most and least important to them regarding lung cancer screening from groups of three. Linear regression was used to determine the association between race and other factors via importance weights [20, 21]. (Figure 3).

Figure 3: Best Worst Scenario Results

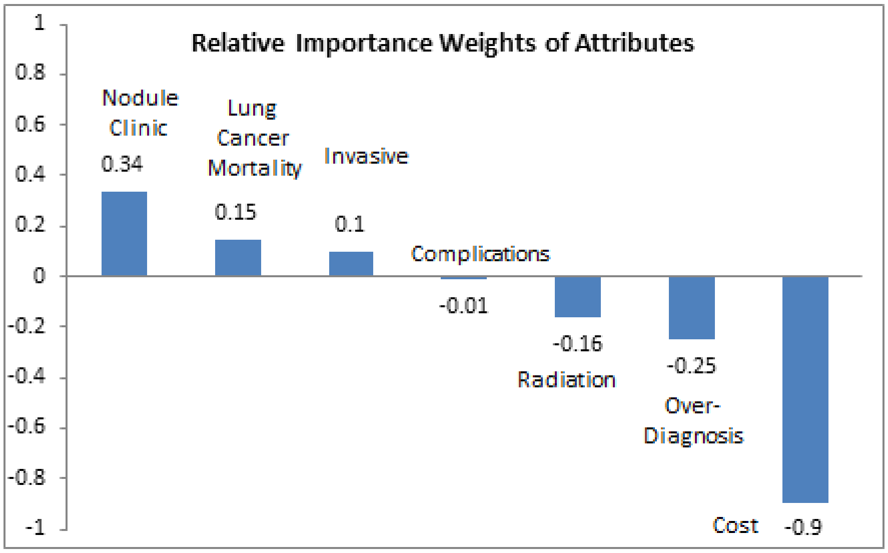

Dr. Schapira also worked with web developers to improve patient understanding of numeric information used in medical diagnosis. Forty-two participants diagnosed with heart failure had the option to learn in a classroom setting, or in a web-based program. The pilot study found that 95% of participants preferred a computer-based tutorial, with the most viewed modules being Understanding Risk and Taking Medications. Knowledge quizzes embedded in the program noted participant knowledge increased after module completion. The pilot studies demonstrate the research team's experience in the field of risk communication, preference assessment, and the development of web-based interventions. The proposed study is a natural extension of this pilot work.

The methods proposed are put forth to help design a Lung Cancer Screening Decision Tool (LCSDecTool) for Veterans. LCSDecTool is intended to improve outcomes and number of high-quality decisions for Veterans eligible for lung cancer screening. High quality decisions are broadly defined as

APPROVED by CMCVAMC IRB on 03/28/2021

informed decisions in which the patient's choice is aligned with their values and preferences. The linkages to outcomes are described in the table below (Table 2).

Table 2: Linkage of Theoretical Constructs to Data Elements, LCSDecTool Design, and Outcome Metrics

Constructs	Data Obtained	LCSDecTool Design	Outcomes
Evidence (PARIHS)	-Patient interviews. Format for presenting evidence, importance of guidelines including smoking cessation (Aim 1).<br>-Provider & clinic leader focus groups. Format of evidence, Importance of guideline recommendations (Aim 1)	Presentation of scientific evidence, (statistics/graphs/prose)<br>Guidelines included (such as ACS, USPSTF, VA, Other)<br>Integration of smoking cessation message	Patient: lung cancer screening knowledge, decision conflict.<br>Providers: satisfaction, usefulness, intention, and actual use of decision tool.
Context (PARIHS)	-Patient interviews. Location and modality (desktop, tablet, phone) of tool use, supporting shared decision making. (Aim 1).<br>-Provider focus groups. Identifying eligible patients, location of tool use, timing of use, supporting shared decision making. (Aim 1).	Referral process for tool, location of tool use, timing of tool use in relation to clinical visit, how summary sheet shared with provider	Patient completion of decision tool,<br>Patient and provider satisfaction with decision tool.
Facilitation (PARIHS)	-Patient interviews. Views about training and facilitation (Aim 1)<br>-Provider focus groups. Views about training and facilitation (Aim 1).	Training of staff, patients, and providers for tool<br>Support services needed to integrate tool in clinic setting	Completion of tool<br>Shared Decision Making occurring in clinic visit.<br>Patient & provider satisfaction with tool.
Usability, Feasibility, Acceptability (TAM)	-Patient interviews. Length, navigation elements, and look and feel of tool; priorities for modalities (phone, tablet). (Aim 1)<br>-Provider focus groups and workshop sessions with web developers. Length of tool, time to complete, modality (tablet, phone, computer), print version (Aims 1, 2).	Look and feel of tool<br>Navigation of tool<br>Length of tool<br>Focus on print version<br>Final version of best worst scenario value elicitation exercise	Time to complete tool<br>Patient engagement<br>End user satisfaction<br>Overall satisfaction
Shared Decision Making (SDM) Framework	-Patient interviews. Values most important to patients, review and possible expansion of attributes used in decision tool; specific content of decision tool (Aim 1)	-refine preference assessment best worst scaling exercise<br>-finalize content in decision tool	Decisional conflict<br>Decision regret<br>Screening uptake<br>Screening adherence

The decision regret scale was adapted to lung cancer screening Data Collection Instrument). Anxiety, as measured by the STAI, and cancer worry have been used as patient-centered outcomes among lung cancer screening recipients [12, 85, 86]. A systematic review reports short-term psychological discomfort among many lung cancer screening recipients and short-term increases in distress among those with false-positive results [87]. The lung cancer worry scale is adapted from a measure of breast cancer worry after mammography screening [88]. A post-visit provider survey will assess whether lung cancer screening was discussed at the visit, if a recommendation was made, and if a test ordered.

The following objectives were completed in Phase 1 and 2.

> ***Phase 1**: To elicit stakeholder input to inform the development of a lung cancer screening decision tool (LCSDecTool). In this Phase we use structured interview and focus groups among patients, providers, and clinical leaders. Findings from Phase 1 were used to develop the LCSDecTool in Phase 2.*
>
> ***Phase 2**: To develop a web based LCSDecTool that will expand existing materials available for Veterans through a Web application that can be used on a desktop, laptop, tablet, or mobile phone. Outcomes will include measures of feasibility, perceived utility, patient engagement, and satisfaction.*

APPROVED by CMCVAMC IRB on 03/28/2021

APPROVED by CMCVAMC IRB on 03/28/2021

Section 4: Objectives Section

4.1. **Describe the study's purpose, specific aims, or objectives.**

Phase 3*: To test the efficacy of the LCSDecTool compared to usual care on improving decision process, clinical, and behavioral outcomes. Primary outcomes include decisional regret, decisional conflict, state trait anxiety, lung cancer worry, and lung cancer screening uptake. We hypothesize that Veterans randomized to the LCSDecTool compared to the control intervention will demonstrate the following:*

- *H1: Decreased decisional regret and decisional conflict at 1 month following the intervention*
- *H2: Decreased state anxiety and lung cancer worry at 1 month following the intervention*
- *H3: Decreased rates of lung cancer screening at 3 months following the intervention*

This work builds directly upon a recently completed HSR&D supported pilot study in the area of lung cancer and shared decision-making. We will accomplish these Aims through a mixed methods study at the CMCVAMC,the WHVAMC, and the Milwaukee VAMC. The study utilizes the expertise of a transdisciplinary research team culminating in a randomized trial to evaluate the efficacy of the LCSDecTool as well as feasibility and process outcomes to support future wide spread implementation. As a new paradigm of lung cancer control is emerging that incorporates both lung cancer screening and smoking cessation, it is imperative to develop a patient centered strategy that incorporates Veterans' values into lung cancer screening decisions that can be integrated into the clinical setting. The proposal provides a mechanism to accomplish these goals and fills a gap in currently available decision support tools for Veterans. The proposed research will augment ongoing efforts by the VHA National Center for Health Promotion and Disease Prevention to promote a patient centered approach to lung cancer screening.

4.2. **State the hypotheses to be tested.**

We hypothesize that Veterans randomized to the LCSDecTool compared to the control intervention will demonstrate the following:

- *H1: Decreased decisional regret and decisional conflict at T2 (1 month after the intervention)*
- *H2: [Decreased overall anxiety and lung cancer worry at T2 (1 month after the intervention)]*
- *H3: Lower rates of lung cancer screening at T3 (3 months after the intervention)*

Section 5: Study Procedures

5.1. **Study Design**

5.1.1. **Describe in detail the experimental design, i.e. from recruitment procedures to study closure.**

A pilot randomized clinical trial (RCT) will be conducted to compare the efficacy of the LCSDecTool to a control intervention among with respect to decision process outcomes, clinical outcomes, and health related quality of life.

Eligible patients will be identified through VA Corporate Data Warehouse (CDW), flyers in primary care/pulmonary clinics and the use of a study team consult that will allow providers to refer eligible patients to the study team. When a potential participant is identified, the study team will send the patient an invitation letter to join the study. Use of the CDW database will support this approach, as the dates of scheduled visits are included in the CDW database and updated daily. Efforts will be made to recruit at least 40% African American, 5% Hispanic, and 10% female patients. Eligibility will be determined by in-person or telephone interviews.

APPROVED by CMCVAMC IRB on 03/28/2021

Since MyHealtheVet will be used after consent, the research team will ask patients if they are registered on MyHealtheVet with a premium account that allows them to access Secure Messaging. If they are not registered, the study team will offer that they go to the MyHealtheVet office to sign up. The study team may use MyHealtheVet Secure Messaging to confirm and schedule research appointments and follow-ups. The use of MyHealtheVet is optional. Patients will be enrolled prior to upcoming appointments in primary care/pulmonary clinics to ensure an opportunity for shared decision-making following the intervention

All primary care providers in the PACT at the respective VA centers will be sent a description of the overall study through email and/or office mail. Providers will be able to opt out of the study, in which case their patients will not be approached regarding study enrollment. For providers who agree to participate, their patients will be approached regarding study enrollment if they meet inclusion and exclusion criteria. A provider information page will be sent to all participating providers which will include information about the LCSDecTool and example of the LCSDecTool summary sheet. This may include the same providers that participated in Phase 1 and Phase 2.

The LCSDecTool will be programmed to randomly assign the participant to 1 of 2 viewing experiences (Experimental vs. Control). The study visit will occur either in-person or by telephone. The in-person study visit will be in a quiet, private room. Telephone visits will be arranged so that coordinators and participants will both be in a quiet, private setting. After completing informed consent and HIPAA Authorization (for baseline in-person visit), the patient will sign on to the study site, complete baseline assessments, and be randomly assigned to the Experimental or Control intervention. Randomization will occur at the patient level. A research assistant will be available to answer questions for participants during the study visit. The visit may be observed by a member of the web development team to better understand how the tool functions.

***Experimental Intervention**: Participants randomized to the experimental intervention will receive a message on the screen that they are now going to view the LCSDecTool. At the end of the tool experience, they will be given the option to print out a summary sheet. Participants will complete the immediate post-intervention assessments (T1) on a computer interface.*

***Control Intervention**: Participants will view screen content that that provides general information on health promotion and disease prevention unrelated to lung cancer screening. No printout is given to subjects in this arm. The control intervention will have the same number of screens to review as the LCSDecTool. Participants will complete the immediate post-intervention assessment (T1) using a computer interface. The Control group will complete all of the surveys, with modified versions of Practical Utility of LCSDecTool, Pt Engagement, and End User Satisfaction that cover general cancer screening rather than Lung Cancer specifically.*

The baseline visit (T0) research visit will last ~2 hours
The immediate post-intervention visit (T1) done in person or by phone will last ~1 hour.

The summary sheet that is presented at the end of the LCSDecTool will have the option to be downloaded, emailed, or printed and a copy given to the subject to have available to refer to during an in-person clinic visit or telehealth visit and serve as a prompt for shared decision-making. The subject or study team will also send the summary sheet to the provider through MyHealtheVet Secure Messaging. Studies suggest that printed action plans can be effective in supporting shared decision-making for collaborative goal setting in primary care. [83] Note, that if the subject chooses use email, the subject will type in their own email address. The study will not be storing that email information in the application back end.

Participants in the control group will NOT be given any materials to bring to their clinical appointment

APPROVED by CMCVAMC IRB on 03/28/2021

Outcome assessment will be blinded to group assignment (Table 4). Primary outcomes include decisional conflict, decisional conflict, general anxiety, lung cancer worry, and lung cancer screening uptake at T1 (1 month post-intervention). Health related quality of life and feasibility measures will also be assessed (Table 4)]. Patient measures will be obtained at baseline (T0), immediately post-intervention (T1), 1 month post-intervention (T2), and 3 months post-intervention (T3). The T2 and T3 survey assessments will be assessed by a telephone interview. At the conclusion of the study, we will mail summary findings of the study to participants.

Provider participation in this study
Healthcare providers participating in this study will undergo oral consent prior to recruiting patients.

For those patient-provider dyads in which the patient was randomized into the intervention, their patient may bring the LCSDecTool to the appointment so that they can review it with the provider. Their patient may also bring their summary page to be reviewed with the provider. The subject or study team will also send the summary sheet to the provider through MyHealtheVet Secure Messaging, for those randomized to the experimental arm. They will have the opportunity to engage in shared decision-making regarding Lung Cancer Screening. For those patient-provider dyads in which the patient was randomized into the control, they will have the opportunity to engage in shared decision making regarding general cancer screening and prevention. For either randomized group, the provider will complete a one page "Post-Visit Provider Survey" after each appointment with participating patients. Each participating provider will have between 0 and 20 patients enrolled in the study. At the conclusion of the study, every provider will complete a 3-page survey, which includes System Usability Survey, Technology Acceptance Model, and End User Computing Satisfaction. Total involvement in the study will take approximately 1 hour.

***Power and Sample Size** - We will enroll 200 Veterans in addition to up to 240 Providers in this pilot RCT, a sample size that is feasible to enroll and that will provide adequate power to permit testing of key study outcomes. We conducted our power analysis comparing the LCSDecTool to usual care with respect to the two primary outcomes, Decisional Regret and Decisional Conflict, assessed at T2 (1 month post-intervention). We will conclude that the intervention was superior if we reject the null hypothesis of no difference for either or both of these outcomes. Tests will be two-sided and will be conducted using α=0.025 to control the family-wise type I error rate at 0.05. Assuming 10% loss to follow-up at one month post-intervention, 180 patients will be available for analysis. For Decisional Regret at T2, we will have 96% power to detect a medium effect size of 0.60 corresponding to a 10 point difference between intervention and control groups. Assuming SD = 16.6 [95]. For the Decisional Conflict outcome, we will have 96% power to detect a medium-sized standardized effect size of 0.5, corresponding to a difference of 2.0 points between groups assuming SD = 3.9. In exploratory analyses of Africa. We will conclude that the intervention was superior if we reject the null hypothesis of no difference for either or both of these outcomes. Tests will be two-sided and will be conducted using α=0.025 to control the family-wise type I error rate at 0.05. Assuming 10% loss to follow-up at one month post-intervention, 180 patients will be available for analysis. For Decisional Regret at T2, we will have 96% power to detect a medium effect size of 0.60 corresponding to a 10 point difference between intervention and control groups, an American participants, we will have 80% power to detect a standardized effect size of 0.74 or larger in either outcome.*

APPROVED by CMCVAMC IRB on 03/28/2021

Table 4: Baseline and Outcome Patient Measures

Variable	T0	T1	T2	T3	Description
SES	X				Age, gender, race, ethnicity, income
Smoking Hx	X				Years of smoking, packs per day, quit year, quit attempts
Health literacy, numeracy	X				Rapid Estimate of Adult Literacy in Medicine (REALM [89]); Numeracy Understanding in Medicine (NUMi) short form [90].
LC screening knowledge	X	X	X	X	12 item T/F scale with the following domains: Eligibility Criteria, Test Interval, Risks and Benefits
Time Discounting Preference	X				How well does this statement describe you as a person? "I give up things today so that I will be able to afford more tomorrow." Use a scale of 0 to 10 where 0 means "does not describe me at all" and 10 means "describes me perfectly" [39]
Intuitive Reasoning	X				Rate the extent that you made this choice based on intuition "by the gut" vs reasoning. Response 1 (only intuition) vs. 9 (only reason) [38]
Mood Assessment	X				10 Items from PANAS-X: sad, happy, angry, nervous, enthusiastic, jittery, hostile, excited, lonely, content (1-not at all to 7-a lot)[91]
Decision Conflict		X	X	X	16 Items, 1 (low)-5 (high), Subdomains: uncertainty, uninformed, unclear values, support, ineffective decision making[92, 93]
Decision Regret		X	X	X	5 item scale, 1 (strongly agree) to 5 (strongly disagree; 0 (no regret) to 100 (high regret). Effect size: 5 pts. Mean (SD): 15.1 (16.5) n=43 to 11.7 (11.8) n=35 [94, 95]. Adapted for lung cancer screening.
State Trait Anxiety Index **	X	X	X	X	STAI 20 items (State) and 20 items (Trait). 4-point scale (Not at all) to (Very much so)[85].
Lung Cancer Worry Interference Scale	X	X	X	X	Adapted from Breast Cancer Worry Scale[88]. 7 item scale, each assessed on a five-point likert sale (Range of scores of 7-35).
Quality of Life- SF-36 v. 2.0	X		X	X	36 items. Domains: physical functioning, role-physical, bodily pain, general health scales, vitality, social functioning, role-emotional, and mental health scales. Scores 0-100.
LC Screening Decision		X	X	X	Main analysis at T3 defined by LDCT obtained within 6 months.
Future Screening Intentions		X	X	X	Intentions to adhere to follow up and future screens. Response scale: 1) Extremely unlikely, 2) Unlikely, 3) Neutral, 4) Likely, 5) Extremely Likely.
Screen completion				X	Yes (completed original scan and follow-up at 6 months)
LC Screening Self-Efficacy	X	X	X	X	Adapted from 10-item self-efficacy for breast cancer screening. Domains: transportation, communication, expected outcomes, scheduling [96]:
Motivation to Quit Smoking	X	X	X	X	Readiness to Quit Ladder: 1 (I enjoy smoking and have decided not to quit smoking for my lifetime. I have no interest in quitting.) to 10 (I have quit smoking and I will never smoke again) [97].
Perceived risk of lung cancer	X	X	X	X	How likely is it that you will get lung cancer? Responses on a scale of no chance (0%) to certain (100%) [85].
Feasibility		X			Time to complete LCSDecTool. Successful completion (yes/no).
Practical Utility of LCSDecTool *		X			9 items (Likert scale 0-10) with domains (Practical Value, Self-Discovery) [98].
Pt Engagement *		X			4 Items, Likert scale 1-5 [98]
End User Satisfaction *		X			12 Items (Yes/No): Content, accuracy, format, ease of use, timeliness. [82, 99, 100],
Time to complete		X			Time to complete LCSDecTool (minutes).
Overall Satisfaction		X			How satisfied were you with this tool? 1: Not at all to 6: extremely satisfied.
LC Shared Decision Making		X			Collaborate, 3-items, patient reported: effort made to 1)help you understand lung cancer screening, 2) listen to the things that matter most to you about lung cancer screening, 3) include what matter most to you in making this choice [101]

Note: T0 is pre-intervention, T1 directly post-intervention, T2 1 mo. post-intervention, T3 3 mo. post-intervention.

* Control group will complete survey pertaining to general cancer screening

**** Only Questions 1 - 20 to be completed for T1, T2, T3**

Amendment February 2019

The purpose of this amendment is to speak to interactions with MyHealtheVet as written in the grant. MHV letter of support attached. In addition, we wanted to add a comment regarding sharing a summary of findings with the patients. There is a small edit to the Post visit provider survey regarding the web based LCS Tool. Last, we changed the scaling on the EUCS survey to mirror the original widely used survey. We removed (6 not applicable from the survey)

Amendment March 2019

The purpose of this amendment is to make minor changes to the post-visit provider survey, future screening intentions and replace the former table 4 with the above.

Amendment April 2020 – addition of the Milwaukee VA site

Veteran recruitment at the West Haven site was low and recruitment at that site will. We are adding the Milwaukee site to try to reach our original goals for patient recruitment. However, each site involves essentially all the primary care providers at the site - hence there will be

another 80 providers recruited from Milwaukee

Amendment September 2020
Due to COVID-19 and the increasing amounts of primary care telehealth appointments we are modifying our protocol to include telephone baseline visits to meet our recruitment goals.
In addition, we are modifying our protocol to increase compensation for follow-up telephone visits for participants at the Philadelphia site. The reasons for increased compensation are to increase follow-up participation and to properly compensate Veterans for their time spent answering survey questions for the study as follow-up calls are taking up to sixty minutes to complete. Currently, we provide Veterans with a $25 stipend after completion of both 1- and 3-month follow-ups. In this modification, we will provide Veterans with $25 stipends for each of the telephone visits. With the modification, the total amount participants receive will increase from $75 to $100.

5.1.2. **What research methods will be used in the project*? Check all that apply.***

- [x] **Surveys/Questionnaires**
- [x] **Interviews**
- [ ] **Audio Taping**
- [ ] **Behavioral Observations**
- [x] **Chart Reviews**
- [ ] **Video Taping**
- [ ] **Focus Groups**
- [x] **Randomization**
- [ ] **Double-Blind**
- [x] **Control Group**
- [ ] **Placebo**
- [ ] **Withhold/Delay Treatment**
- [ ] **Specimen Collection**
- [ ] **Deception**
- [x] **Telephone Survey**
- [ ] **Other (Describe)**

5.1.3. **Provide description of the study population (delineate all categories of subjects – male, female, inpatients, outpatients, providers, family members, employees, etc.). Include anticipated initial enrollment numbers (and number of subjects anticipated to complete all aspects of the protocol).**
This project involves the recruitment of 200 Veterans and up to 240 Providers. Patient recruitment efforts at the three sites (CMCVAMC, WHVAMC, and Milwaukee VAMC) will target male and female patients enrolled in a Patient Aligned Care Teams (PACT) who are eligible for lung cancer screening. Our recruitment goals for patients are 40% African American, 5% Hispanic patients, and 10% women.

5.1.4. **As applicable, provide rationale and information on any added protections and safeguards for vulnerable populations (children, prisoners, pregnant women, physically or mentally-disabled persons, and economically or educationally disadvantaged persons).**
N/A

5.1.5. Does this project target a specific race or ethnic group as subjects? *Yes.* **If <u>yes</u>, check all that apply.**

<u>Race</u>

- [ ] **American Indian or Alaska Native**
- [ ] **Asian**
- [x] **Black or African American**
- [ ] **Native Hawaiian or other Pacific Islander**

<u>Ethnicity</u>

- [ ] **Hispanic or Latino**
- [ ] **Not Hispanic or Latino**

APPROVED by CMCVAMC IRB on 03/28/2021

☐ **White**

☐ **Other** ☐

5.1.6. **Will this study bank/store specimens for future research?** *NO*

NOTE: Banking is for future specified research. Storage is for the life of this protocol.

5.1.6.1. **If yes, include information on specimens to be banked/stored.**

5.1.6.2. **If specimens will be banked/stored, specify location.**

5.1.6.2.1. ***IF BANKING SPECIMENS, IT MUST BE AT A VA APPROVED FACILITY. (For additional information, go to the following website http://www.research.va.gov/programs/tissue_banking/, or contact the IRB office.)***

5.1.6.3. **If applicable, explain how destruction of banked samples will be substantiated.**

5.1.6.4. **Do you anticipate using the banked specimens for other studies beyond the defined study period and defined study parameters?** *Choose an item.*

5.1.6.4.1. **If yes, will you need to re-contact subjects? How will this be done?**

5.1.7. **Will this study create a data repository for future studies?** *NO*

5.1.7.1. **If yes, describe and/or provide the following:**

5.1.7.1.1. **The type of data (identified or de-identified) including what protected health elements are to be collected.**

5.1.7.1.2. **The source from which data will be collected (e.g., subjects, non-research data repositories, research data repositories, publicly available, VA source, non-VA source.**

5.1.7.1.3. **How and where the data will be stored (e.g., electronic, paper records, approved VA-owned or VA-leased space).**

5.1.7.1.4. **How the data will be transmitted, if applicable.**

5.1.7.1.5. **How the data will be secured during storage, use, and transmission both during the conduct of the research protocol and after the protocol is completed.**

5.1.7.1.6. **Plans to store data for future research. If the data is stored for future research, there must be a description of a research data repository, its location, and its security measures.**

5.1.7.1.7. **Plans to share with others including other researchers (VA and non-VA). If the data were collected through a research project, discussion of whether or not the original informed consent allowed for such reuse of the data and if the reuse is consistent with the HIPAA authorization that was obtained.**

APPROVED by CMCVAMC IRB on 03/28/2021

5.1.7.1.8. **Justification for the use of any identifiers.**

5.1.7.1.9. **Justification that the data requested represent the minimum necessary to conduct the research.**

5.1.7.1.10. **A discussion of plans for obtaining informed consent and HIPAA Authorization, or for requesting the IRB to waive these requirements. If the investigator requests that the requirement for a HIPAA Authorization be waived, justification for this request must be included in information submitted to the IRB.**

5.1.7.1.11. **In addition to the above, provide a Standard Operating Procedures Manual for the data repository.** *Contact IRB office for additional details.*

(*NOTE: The investigator is encouraged to consult the facility's ISO during the development of the protocol to ensure compliance with all information security requirements and physical security requirements.)*

5.2. **Subject Recruitment Methods**

NOTE: Details of recruitment should be included in the 'procedures' section. Only 'short' responses should be included in the below sections.

5.2.1. **State how many subjects will be needed**.

This project involves the recruitment of up to 100 Veterans and 80 providers from the CMCVAMC/CBOCS.

This project involves the recruitment of up to 200 Veterans between CMCVAMC/CBOCS, WHVAMC, and Milwaukee VAMC. This project also involves the recruitment of up to 240 Providers (80 from each site).

5.2.2. **Who will be responsible for recruiting potential subjects**? **Provide titles of individuals.**

Principal Investigator, Sr. Coordinator, and the Research Assistant

5.2.3. **How will initial contact with potential subjects be made? (*e.g., local clinics, physician referrals, letters to prospective subjects*)**

NOTE: VA policy prohibits "cold calls" to potential VA research subjects. Provide an introductory letter and telephone script.

Eligible Veterans will be mailed a recruitment letter with study information and an invitation to participate. A study staff member will make a follow-up phone call to the Veteran if there is no reply after one week. During screening phone call, inclusion/exclusion criteria will be confirmed. A second recruitment strategy will be to post study flyers and brochures in the primary care and pulmonary clinic setting and have a recruitment table in the waiting room to provide study information. Study visits will be scheduled the same day as clinic appointments when possible. If necessary, due to the timing, participants can be scheduled up to 2 weeks prior to their appointment time .

5.2.4. **Will you be using any of the following methods to recruit subjects? (Check all that apply.)**

☐ **N/A**

☑ **Local database for which subjects have NOT given prior permission to be contacted for Research.**

APPROVED by CMCVAMC IRB on 03/28/2021

NOTE: If this option is checked, please submit a Waiver of Individual Authorization for Disclosure of Protected Health Information.

☐ **Personal contact with patients over whom you have direct/indirect oversight**

☑ **Provider (Clinician) Referrals of potential subjects**

5.2.5. **Indicate the types of recruitment/advertisement materials that will be used: Check all that apply. Submit copies of recruitment materials, for IRB review**.

☐ **Not applicable; none to be used**

☐ **Fliers** ☐ **Newspapers** ☑ **Letters** ☐ **Websites** ☐ **Television**

☐ **Radio** ☐ **Audio** ☐ **Video** ☐ **Surveys**

☑ **Other (Specify, e.g. employee newsletters**) *Study Brochure, Emails to Providers*

5.2.6. **Non-Veteran Subjects will be given a copy of the Notice of Privacy Practices.** *YES*

NOTE:

- *Every non-Veteran should sign VA form 10-0483, Acknowledgement of the Notice of Privacy Practices (ANOP)*
- *Once the ANOP is signed, the research study staff must send the non-Veteran's name to the CMCVAMC Privacy Officer via encrypted e-mail. The signed ANOP must be kept in the research study binder.*
- *If an oral informed consent is used, the NOP should be sent to the non-Veteran via postal mail. In addition, the research study staff must write a Note-to-File that the NOP was sent to the non-Veteran.*

5.3. **Compensation for Participation - ** *YES.* **If yes, complete the following**.

5.3.1. **Summarize any financial compensation that will be offered to subjects**.

Veterans will be compensated $100 total incentive ($50 for completing the baseline and immediate post-intervention surveys; and $25 each for completing the 1 and 3-month telephone follow-ups).

5.3.2. **Provide the schedule for compensation**.

5.3.2.1. **Per study visit or session.**

$50 for completing the baseline and immediate post-intervention surveys, $25 for completing the 1-month telephone follow-up, and $25 for completing the 3-month telephone follow-up.

5.3.2.2. **Total amount for entire participation.**

$100 total incentive

5.3.3. **State how compensation will be provided:** *In-person visits– voucher given in personTelephone visits – vouchers given to research budget office who mails participant the checks.*

5.4. **Informed Consent Procedures**

5.4.1. **Indicate if informed consent will be obtained and/or if you are requesting a waiver of informed consent or waiver of documentation of informed consent.**

Consent to be obtained at baseline; written consent for Veteran with in-person baseline visits; oral consent for Veteran telephone visits. Oral consent for providers.

5.4.2. **If the research involves multiple phases, specify for which phases of the research the waiver(s) is/are being requested.**

HIPAA waiver will be obtained to screen computer databases for potential Veteran

APPROVED by CMCVAMC IRB on 03/28/2021

subjects who will be contacted to see if they are interested in participating in research. HIPAA waiver is not needed for Provider subject because study team is NOT collecting patient PHI from the provider.

5.4.3. **Describe circumstances, if any, that may need to be addressed in seeking informed consent (e.g., subjects with impaired decision-making ability and the use of a legally authorized representative, etc.)**

NA

5.4.4. **If applicable, indicate how study personnel will be trained regarding human subjects protections requirements and how to obtain and document informed consent.**

All study personnel will be trained/overseen by PI. They will have completed relevant training regarding human subjects protection and how to obtain informed consent. All personnel are required to have CITI (Good Clinical Practices and Ethical Principles), VA Privacy and Information Security Awareness and Rules of Behavior, and Privacy and HIPAA trainings. In addition, all study personnel obtaining informed consent will have taken the VA Research Compliance Officer training.

5.4.5. **Inclusion/Exclusion Criteria: Describe the criteria that determine who will be included in or excluded from the study.**

5.4.5.1. **Inclusion Criteria**

All Veterans must be enrolled in a Patient Aligned Care Teams (PACT) who are eligible for lung cancer screening. Veterans will be age 55 to 80 years, smoking history of 30 or more pack years, and current smoker or smoker who quit within 15 years whose providers have agreed to also participate in the study

Inclusion criteria for providers will include practicing or providing leadership in a PACT or involvement in the lung cancer screening through the clinical services of Radiology, Oncology, or Pulmonary Medicine, services that are most involved with the lung cancer screening.

5.4.5.2. **Exclusion Criteria**

Exclusion criteria for Veterans will be cognitive impairment as determined by clinical history, previous diagnosis of cancer with the exception of non-melanoma skin cancer and localized prostate cancer that is 1-year post-diagnosis or more and with no indication of progression, active surveillance of Lung Nodule, Enrolled in CMCVAMC Lung Cancer Screening Program, life expectancy of less than 2 years as indicated by chart review and confirmation with PCP, and inability to speak English during the phone screen.

5.5. **Withdrawal of Subjects**

5.5.1. **Describe how a subject can withdraw from the study**.

Veteran subjects who want to withdrawal from the study should do so using the HIPAA Revocation form. Providers who want to withdrawal from the study may do so verbally.

5.5.2. **Describe any anticipated circumstances under which subjects will be withdrawn from the research without their consent**.

NA

5.5.3. **Describe the consequences of a subject's decision to withdraw from the research and the procedures for orderly termination of participation by the subject (e.g., the subject contacting the investigator for an end-of-study visit)**.

APPROVED by CMCVAMC IRB on 03/28/2021

No consequences for withdrawal. Once we are notified of a participant's decision to withdraw, we can continue to use information about them that has been collected up to that point. No information will be collected after a participant formally withdraws

5.6. **Potential Risk/Benefit Analysis**

5.6.1. **Potential Study Risks**

5.6.1.1. **Describe and assess all of the following risks that may be associated with the research**:

5.6.1.2. **Physical** (*Physical risks include physical discomfort, pain, injury, illness or disease brought about by the methods and procedures of the research. A physical risk may result from the involvement of physical stimuli such as noise, electric shock, heat, cold, electric magnetic or gravitational fields, etc. Engaging a subject in a social situation which could involve violence may also create a physical risk*.)

NA

5.6.1.3. **Psychological** (*Psychological risks include the production of negative affective states such as anxiety, depression, guilt, shock and loss of self-esteem and altered behavior. Sensory deprivation, sleep deprivation, use of hypnosis, deception or mental stress are examples of psychological risks.)*

The Veteran subjects may risk distress from considering the topic of lung cancer. Veterans in the study will be at increased risk for lung cancer due to the smoking exposure eligibility requirements. Therefore, it is possible that a discussion of lung cancer screening may lead to anxiety or distress. Subjects with anxiety or concerns will be referred to their primary care physician as all subjects are enrolled in a primary care clinic as it is an inclusion criterion for the study. The primary care physician will be able to evaluate Veteran's level of anxiety/distress and refer them to mental counseling if needed.

Provider and clinical leader participants may risk anxiety regarding the potential implementation of a new clinical procedure (Lung cancer screening shared decision-making) in a busy clinical practice.

5.6.1.4. **Social/Economic** (*Social/Economic risks include alterations in relationships with others that are to the disadvantage of the subject, including embarrassment, loss of respect of others, labeling a subject in a way that will have negative consequences, or in some way diminishing those opportunities and powers a person has by virtue of relationships with others. Economic risks include payment by subjects for procedures not otherwise required, loss of wages or other income and any other financial costs, such as damage to a subject's employability, as a consequence of participation in the research.*)

NA

5.6.1.5. **Legal** (*Legal risks exist when the research methods are such that the subject or others will be liable for a violation of the law, either by revealing that the subject or others have engaged, or will engage, in conduct for which the subject or others may be criminally or civilly liable, or by requiring activities for which the subject or others may be criminally or civilly liable.)*

NA

5.6.1.6. **Loss of Confidentiality** *(In all research involving human subjects, confidentiality of identifiable information is presumed and must be maintained unless the investigator obtains the express permission of the*

APPROVED by CMCVAMC IRB on 03/28/2021

subject to do otherwise. Subjects have the right to be protected against injury or illegal invasions of their privacy and to preservation of their personal dignity. The more sensitive the research material, the greater the care that must be exercised in obtaining, handling, and storing data. In order to minimize the risk for loss of confidentiality, investigators should only collect personal information that is absolutely essential to the research activity. If personal data must be collected, it should be coded as early in the activity as possible and securely stored so that only the investigator and authorized staff may access it. Identities of individual subjects must never be released without the express consent of the subject. In addition, if an investigator wishes to use data for a purpose other than the one for which it was originally collected, and the data are still identifiable (e.g., a code list for the data still exists), the investigator may need to obtain consent from the subjects for the new use of the data.)

There is some risk of confidentiality. Efforts will be made to minimize this risk by collecting only information that is absolutely essential to research. Veteran's protected health and personally identifiable information will be coded and securely stored in a file cabinet behind locked doors in CHERP Annex Room 232. Identified and coded data will be kept in two separate file locations, both located in Room 232. The data collected will only be used for the purposes of the study protocol

5.6.1.7. **Other, e.g. radiation, placebo, washout of medications**

NA

5.6.1.8. **Assess the likelihood and seriousness of such risks**.

The risk of Veterans feeling distress about discussing cancer screening is not likely to be serious since this targeted group would have most likely discussed lung cancer screening with their providers. The risk of clinicians/leaders' facing negative social opinions is highly unlikely since participants will be working together and inputting ideas. Theft, loss and unauthorized access will be reported immediately to the Privacy Officer, Information Security Officer and IRB

5.6.2. **Include a description of how anticipated risk will be minimized and include an analysis of risk vs. potential benefit.**

Veterans with anxiety or concerns will be referred to their primary care physician. Each Veteran will be assigned an ID number that is not related to any PHI. The providers/leaders will be assigned an ID number so that their baseline assessment can be linked to an ID number. The key containing the link between the study identification number and participant will be maintained on the CMCVAMC's secure server (\\vhaphifpccherp.v04.med.va.gov\shares2\Schapira_Marilyn) on a separate fold from the collected data. Only Dr. Schapira (PI), Research Coordinator, and Research Assistant will have access to this key.

5.6.3. **Potential Study Benefits**

5.6.3.1. **Indicate potential benefits to be gained by the individual subjects, as well as benefit(s) that may accrue to society in general as a result of the planned work. If the subject will not receive any direct benefit, this fact must be stated here and in the consent form.**

The study may not benefit participants. It could benefit future Veterans if a patient centered approach to decision-making regarding lung cancer screening works. This may benefit Veterans and others who are considering

APPROVED by CMCVAMC IRB on 03/28/2021

lung cancer screening and would like a structured way to assess whether lung cancer screening is good option for them

5.6.4. **Alternative Treatments Outside the Study**

5.6.4.1. **Describe alternatives available to the subject outside the research context. If there are no such alternatives, state that the alternative is not to participate in the research study**.

This is not a treatment study. The alternative is not to participate.

5.7. **Data Monitoring** (*Monitoring plans describe how study records are inspected to ensure the study is adhering to the study protocol and applicable research regulations and CMCVAMC requirements. Monitoring plans do not necessarily require the use of an independent Data and Safety Monitoring Board (DSMB). Such independent boards are usually reserved for high-risk phase I studies, or large, multi-center phase III trials. Federally funded studies may require the use of an independent DSMB.)*

5.7.1. **Will a Data and Safety Monitoring Board (DSMB) or Data Monitoring Committee (DMC) oversee the project?** *YES*

5.7.1.1. **If yes, provide contact information for the DSMB or DMC representative**.

A data and safety monitoring committee will be formed and include Dr. Chhatre (Co-I) and Dr. Hubbard (biostatistics). The committee will report to the investigative team on a monthly basis. The committee will review the accuracy and integrity of the data and monitor adequate subject recruitment and enrollment. Dr. Hubbard (biostatistics) is experienced in data management methods. This committee will review all reports of adverse events within 24 hours of their occurrence and on a monthly basis and determine if a change in protocol is indicated due to the occurrence of adverse events

5.7.1.2. **If no, describe the data and safety monitoring plan to be followed.**

5.8. **Reporting of Protocol Deviations, Adverse Events (AEs), Serious Adverse Events (SAEs), Breaches of Confidentiality, Unanticipated Adverse Device Effects (UADEs), and Unanticipated/Unexpected Problems**

5.8.1. **Include procedures for reporting these events to the CMCVAMC IRB and sponsor**. *NOTE: Except for AEs, all other events must be reported to the CMCVAMC IRB within 5 business days of discovery. Use the CMCVAMC Serious-Adverse Event form for reporting SAEs, UADEs, and unanticipated/unexpected problems. Use the CMCVAMC Protocol Deviation form for reporting protocol deviations. On-site AEs should be reported at the time of continuing review.)*

Protocol deviations, unanticipated/unexpected problems will be reported to the IRB within 5 business days of discovery. Since this study involves interviews, survey assessments, and obtaining feedback for the LCSDecTool, we do not anticipate any adverse events or serious adverse events. However, any serious adverse event ***related to this study*** *would be reported to the IRB within 5 business days of discovery. Any adverse event would be reported to the IRB at continuing review*

5.9. **Privacy and Confidentiality**

5.9.1. **Describe whether the study will use or disclose subjects' Protected Health Information (PHI).**

For Veterans, we will need to obtain name, date of birth, complete mailing address, social security numbers, race, gender, and medical history such as smoking history, lung cancer history, and life expectancy

APPROVED by CMCVAMC IRB on 03/28/2021

No medical history will be collected from clinical providers. We will collect information such as name, gender, job title, years in current job, email, phone number for clinical leaders/providers.

5.9.2. **Check the PHI to be collected on all subjects for this research protocol.**

- [x] **Name**
- [x] **All geographic subdivisions smaller than a State, including street address, city, county, precinct, ZIP code, and their equivalent geographical codes, except for the initial three digits of a ZIP code if, according to the current publicly available data from the Bureau of the Census:**
  - **a. The geographic unit formed by combining all ZIP Codes with the same three initial digits contains more than 20,000 people; and**
  - **b. The initial three digits of a ZIP Code for all such geographic units containing 20,000 or fewer people are changed to 000.**
- [x] **All elements of dates (except year) for dates directly related to an individual, including birth date, admission date, discharge date, date of death; and all ages over 89 and all elements of dates (including year) indicative of such age, except that such ages and elements may be aggregated into a single category of age 90 or older.**
- [x] **Telephone numbers**
- [ ] **Fax numbers**
- [x] **Electronic mail addresses**
- [x] **Social Security/Medical Record Number**
- [ ] **Health plan beneficiary numbers**
- [ ] **Account Numbers**
- [ ] **Certificate/license numbers**
- [ ] **Vehicle identifiers and serial numbers, including license plate numbers**
- [ ] **Device identifiers and serial numbers**
- [ ] **Web universal resource locators (URLS)**
- [ ] **Internet protocol (IP) address numbers**
- [ ] **Biometric identifiers, including fingerprints and voiceprints**
- [ ] **Full-face photographic images and any comparable images**
- [ ] **Any other unique identifying number, characteristic, or code, unless otherwise permitted by the Privacy Rule for re-identification.**
- [ ] **HIV (testing or infectious disease) records**
- [ ] **Sickle cell anemia**
- [ ] **Drug Abuse Information**
- [ ] **Alcoholism or Alcohol Use**

5.10. **Information Security** *(Contact the Information Security Officer for additional assistance regarding confidentiality (storage/security) of research data.)*

5.10.1. **List the data/information that will be stored (including signed, original informed consent and HIPAA authorization forms, if applicable, case report forms, etc.)**

Subject log, signed Consent documents, signed HIPAA authorizations, paper surveys and electronic database of survey data

APPROVED by CMCVAMC IRB on 03/28/2021

5.10.2. **Describe the steps that will be taken to secure the data (e.g., training, authorization of access, password protection, encryption, physical controls, Certificates of Confidentiality and separation of identifiers and data)**.

All paper/hard copy data - signed consent and HIPAA authorization forms, subject logs, surveys will be maintained in study binders. The study binders in CMCVAMC will be kept in CHERP, 4100 Chester Ave, Ste 202 in a locked file cabinet. Any electronic data—the subject logs and the key linking participants to their study ID at CMCVAMC will be kept on the secured CMCVAMC server vhaphicherpnas. (\\vhaphifpccherp.v04.med.va.gov\shares2\Schapira_Marilyn) The log and the key will be kept in separate folders on the server and will not be printed out.

Study personnel with access to study data who are no longer involved with the study data will have access revoked and will be removed from the research staff form

5.10.3. **Indicate how and where data/information will be stored and specify pertinent security systems**.

All paper/hard copy data-consent forms, subject logs, HIPAA authorization, surveys will be maintained in study binders. The study binders in CMCVAMC will be kept in CHERP, 4100 Chester Ave, Ste 202 in a locked file cabinet. Study staff will walk the hard copy documents to CHERP from the CMCVAMC after interviews sessions. Any electronic data-- the subject logs and the key linking participants to their study ID--at CMCVAMC will be kept on the secured VA server vhaphicherpnas. The log and the key will be kept in separate folders.

Electronic Survey data will be entered into the VA REDCap database. No Identifiable information will be included with this data. The VA REDCap database is kept on a VA secured server that is only accessible through an application process

5.10.4. **Will PHI be transmitted or transported outside of CMCVAMC?** *NOT APPLICABLE* **If yes, complete sections 5.10.4.1 through 5.10.4.3, and an Off-site Storage/Transfer of Research Data form. <u>If no, go directly to section 5.11.</u>**

5.10.4.1. **Does the informed consent document and Authorization for Use & Release of Individually Identifiable Health Information for Veterans Health Administration (VHA) Research form disclose entities/individuals to which/whom PHI will be transported or transmitted?** *Choose an item.*

5.10.4.2. **Specify entities/individuals outside CMCVAMC to which/whom data will be disclosed, the justification for such disclosure and the authority, and how they will access it**.

5.10.4.3. **List the data/information that will be transmitted or transported, and specify how data will be transported or transmitted from one location to another and how it will be protected during transmission or transportation outside of CMCVAMC.**

5.11. **Data Management Access Plan -** *NOTE: Effective January 1, 2016, all **new** proposals for VA research (regardless of how the research is to be supported) **must** include a Data Management and Access Plan (DMAP) that describes how PUBLICATIONS resulting from the research and the FINAL DATA SETS underlying such publications will be made available to the public.*

5.11.1. DMAP form **must** be included with all **initial** submissions. The DMAP form can be found on the Research and Development SharePoint site https://vaww.visn4.portal.va.gov/philadelphia/home/Research/default.aspx or obtained from one of the IRB Administrators.

APPROVED by CMCVAMC IRB on 03/28/2021

5.12. **Communication Plan**

5.12.1. **Include plan for ensuring that the study is conducted according to the IRB-approved protocol.**

Monthly team meetings will be held with the PI and study staff to discuss the study progress. The PI will ensure that all study activities are being conducted according to the IRB approved protocol

5.12.2. **If a multi-site study, include information on**

- **ensuring that all required local site approvals are obtained and notifying the Director of any facility where the research is being conducted but the facility is not engaged, and**
- **keeping all engaged sites informed of changes to the protocol, informed consent, and HIPAA authorization**, and
- **informing local sites of any Serious Adverse Events, Unanticipated Problems, or interim results that may impact conduct of the study**, and
- **notifying all local facility directors and local site investigators (LSI) when a multi-site study reaches the point that it no longer requires engagement of the local facility (e.g., all subsequent follow-up of subjects will be performed by the PI from another facility).**

Dr. Schapira (PI) will have overall responsibility for the coordination of the study sites, study team members, and the achievement of scientific goals and objectives.

Dr. Bastian, site PI for WHVAMC, will have direct oversight for activities at the WHVAMC. Dr. Schapira and Dr. Bastian will have bi-weekly conference calls to coordinate study protocol and recruitment efforts.

Dr. Whittle, site PI for Milwaukee VAMC, will have direct oversight for activities at the Milwaukee VAMC. Dr. Schapira and Dr. Whittle will have bi-weekly conference calls to coordinate study protocol and recruitment efforts

Dr. Schapira will also have bi-weekly meetings with the analysis team, led by Dr. Hubbard (biostatistics) and including Christopher Roberts (Data Analyst) and Dr. Chhatre to review data collection and recruitment procedures.

Drs. Schapira, Bastian, and Whittle will have quarterly calls with the advisory committee members.

The advisory committee members are Tanner Caverly, MD of Ann Arbor VAMC and Sarah Lillie, PhD of Minneapolis VAMC.

A Data Review and Monitoring Board (DRMB) will be established led by Dr. Hubbard (along with Dr. Chhatre). The DRMB will meet on a monthly basis to monitor all data collection procedures and ongoing data analysis regarding recruitment rates and outcomes at appropriately pre-determined intervals

5.13. **Is this Study Investigating the Use of a Drug or Biological Agent?** *NO* **If yes, complete the rest of this section. If no, go directly to section 6, unless 5.13 applies.** *NOTE: If this study involves an investigational drug, investigator must contact the Pharmacy and Therapeutics (P&T) Committee and provide its approval to IRB.*

5.13.1. **Specify if the drug or biological agent is:**

5.13.1.1. **FDA approved:** *Choose an item.*

5.13.1.2. **Used for off-label purposes:** *Choose an item.*

APPROVED by CMCVAMC IRB on 03/28/2021

5.13.2. **Include the FDA Investigational New Drug (IND) number for all non-FDA approved and off-label drugs, biological agents or nutritional supplements. If not applicable state, "Not Applicable."**

5.13.3. **Provide all relevant information about the drug, including pre-clinical data.**

5.13.4. **Explain any wash-out periods, rescue medications permitted, and any type of medications not permitted while enrolled in the study.**

5.13.5. **Describe blinding and un-blinding procedures.**

5.13.6. **Include the dosage, route of administration, previous use, and the safety and efficacy information on any drug used for research purposes.**

5.13.7. **Describe rationale for the dosage in this study.**

5.13.8. **Justify why the risks are reasonable in relation to anticipated benefits and/or knowledge.**

5.13.9. **Describe where drug preparation will be done.**

5.13.10. **All drugs for CMCVAMC subjects must be dispensed through the VA investigational pharmacy.**

5.13.11. **Describe where the study treatment will be administered.**

5.13.12. **Describe plan for tracking a non-compliant treatment study subject.**

5.13.13. **Describe the process for the storage, security, dispensing and return of an investigational drug.**

5.13.14. **Has this protocol has been submitted to the Medical Center's Pharmacy and Therapeutics Committee?** *Choose an item.*

5.14. **<u>Is this Study Investigating the Use of a Device</u> –** *NOT APPLICABLE* **If yes, complete the rest of this section. <u>If no, go directly to section 6</u>.**

5.14.1. **The Investigational Device Exemption (IDE) number must be submitted for all significant risk devices and if an IDE exists for a non-significant risk device.**

5.14.2. **Significant Risk or Non-significant Risk - If a device is not approved by the FDA, specify whether or not the sponsor has determined this device to be a "significant risk" or "non-significant risk" as defined by the FDA.**

5.14.3. **Provide all relevant information about the device.**

5.14.4. **Describe blinding and un-blinding procedures.**

5.14.5. **Specify if device is:**

5.14.5.1. **FDA approved:** *Choose an item.*

5.14.5.2. **Used for off-label purposes:** *Choose an item.*

5.14.6. **Explain if the investigational device will be delivered and/or stored by the Principal Investigator or Pharmacy Service.**

APPROVED by CMCVAMC IRB on 03/28/2021

5.14.7. **Describe the process for the storage, security, dispensing and return of an investigational device.**

5.14.8. **For research involving an investigational device, describe the SOP or plan for device control.**

5.14.9. **Address how the device will be stored in such a way that only research staff associated with the protocol will have access to the device.**

5.14.10. **Describe measures that will be put into place to ensure that the device will only be used in subjects of this research protocol.**

Section 6: Resources and Personnel

6.1. **Include where and by whom the research will be conducted**.

The study will be conducted by the PI, Co-Investigators, and other trained research staff at the CMCVAMC

6.2. **Provide a brief description of each individual's role in the study. Indicate who will have access to protected health information and who will be involved in recruiting subjects; obtaining informed consent; administering survey/interview procedures; and performing data analysis**.

- *The Principal Investigator will have access to all study data, be involved with recruitment, obtaining informed consent and HIPAA authorization, administering interviews, and performing data analysis.*
- *The Co-Investigators meet monthly to discuss study design and oversee study progress.*
- *The Research Coordinator and research assistants will be involved with recruitment, obtaining informed consent and HIPAA authorization, and administering interview*
- *Collaborator will be involved in qualitative analysis and development of value clarification tool.* ***This person will not have access to PHI****.*
- *Biostatistician will be involved in quantitative data analysis.* ***This person will not have access to PHI***
- *Dissemination Manager will be involved in communication of study findings.* ***This person will not have access to PHI***
- *Dataset Administrator will be involved in data pulls from CDW for recruitment lists. This person will have access to PHI but not involved in recruiting subjects, obtaining informed consent and administering survey/interview procedures.*
- *The Qualitative Researcher will be involved with assisting in qualitative analysis and development of value clarification tool.* ***This person will not have access to PHI****.*

6.3. **If applicable, provide information on any services that will be performed by contractors, including what is being contracted out and with whom**.

Pun'k Ave is a web site developer that has an extensive experience in the development of interactive patient oriented interventions that are integrated into clinical trials of behavioral interventions. The activities conducted by Pun'k Ave as outlined in their letter of support include server set up with coordination with VA IT and HIPAA compliant infrastructure, visual design, development of the basic application framework, incorporation of a randomized questionnaire module, user management and authentication capability, design of a basic administrative module for data reporting, and consideration of user experience and strategy. This contract will include server and technical support during the full course of the study

6.4. **If applicable, provide information on any Memoranda of Understanding (MOUs) or Data Use Agreements (DUAs) that are being entered into, including with whom and for what**

APPROVED by CMCVAMC IRB on 03/28/2021

reason.

NA

Section 7: Genetic Testing

7.1. **Does the project involve genetic testing?** *Not Applicable, SKIP TO SECTION 8*

7.2. **Will specimens be kept for future, unspecified use?** *Choose an item.*

7.3. **Will samples be made anonymous to maintain confidentiality?** *Choose an item.* (*If there is a link, it is not anonymous. Coding is not anonymous.)*

7.4. **Will specimens be destroyed after the project-specific use is completed?** *Choose an item.*

7.5. **Will specimens be sold in the future?** *Choose an item.*

7.6. **Will subjects be paid for their specimens now or in the future?** *Choose an item.*

7.7. **Will subjects be informed of the results of the specimen testing?** *Choose an item.*

7.8. **Are there any implications for family members based on specimen testing results?** *Choose an item.*

7.8.1. **If answer to section 7.8 is yes, they may be subjects**.

7.9. **Will subjects be informed of results obtained from their DNA?** *Choose an item.*

7.10. **Explain if the study is looking for an association between a genetic marker and a specific disease or condition, but at this point it is not clear if the genetic marker has predictive value**.

7.11. **Describe if the study is based on the premise that a link between a genetic marker and a specific disease or condition is such that the marker is clinically useful in predicting the development of that specific disease or condition.**

7.12. **Will the subject be notified of the results and the provision for genetic counseling?** *Choose an item.*

Section 8: International Research

8.1. **Does this study involve international research?** *NOT APPLICABLE* **If no, go directly to section 9.**

NOTE: Refer to VHA Handbook 1200.05 and contact the IRB office for additional guidance.

Section 9: Statistical Analysis

9.1. **Include statistical power calculations and the assumptions made in making these calculations.**

Specific Aim 3 *To test the efficacy of the LCSDecTool compared to usual care on decision process, psychosocial, and behavioral outcomes. We hypothesize that Veterans randomized to the LCSDecTool compared to the control intervention will demonstrate the following:*

- *H1: Decreased decisional regret and decisional conflict at T2 (1 month after the intervention)*
- *H2: [Decreased overall anxiety and lung cancer worry at T2 (1 month after the intervention)]*
- *H3: Lower rates of lung cancer screening at T3 (3 months after the intervention)*

Descriptive Analysis - *Descriptive statistics will include frequencies and percentages for categorical variables, and either mean and standard deviation or median and interquartile range for continuous variables, as appropriate based on distributional characteristics. We will compare baseline characteristics between groups to assess the adequacy of randomization, using t-tests to compare continuous variables and chi-square tests for categorical variables. Characteristics found to differ significantly between intervention groups (defined as $p<0.15$) will be included as covariates in adjusted analyses. We will examine the distributions of decisional regret, decisional conflict, STAI, and lung cancer worry at T2 (1-month post-intervention) graphically and will calculate summary statistics to assess symmetry and substantial departures from normality.*

APPROVED by CMCVAMC IRB on 03/28/2021

9.2. **Define plans for data and statistical analysis, including key elements of the statistical plan, stopping rules and endpoints.**

Statistical Analysis for H1 *- Our primary analysis for H1 outcomes will be intention to treat, including all patients as randomized in the analysis, using linear regression to test whether decisional regret and decisional conflict scores differ by intervention group at T2 after adjustment for baseline characteristics and study site. If the outcome distribution exhibits substantial skew or deviates in other ways from approximate normality, generalized linear mixed models or alternative methods will be used. The dependent variable will be either Decisional Regret or Decisional Conflict, and the primary independent variable will be intervention group. We will include in all models fixed effects for study site to account for potential variation in outcomes associated with care environment. Adjusted analyses will include variables not balanced by randomization. Standard regression diagnostics will be performed and robustness of results to analysis assumptions will be assessed in sensitivity analyses. Tests will be conducted at the two-sided α=0.025 level so that the overall type 1 error rate is controlled at the 0.05 level (Bonferroni adjustment).*

Statistical Analysis for H2 *- Our analysis for H2 outcomes will be intention to treat, including all patients as randomized in the analysis, using linear regression to test whether STAI and lung cancer worry differ between interventions at T2 after adjustment for baseline characteristics and study site. The approach de-scribed above for H1 will be used, with outcomes defined as STAI and lung cancer worry at T2. Standard regression diagnostics will be performed and robustness of results to analysis assumptions will be assessed in sensitivity analyses. Tests will be conducted at the two-sided α=0.025 level so that the overall type 1 error rate is controlled at the 0.05 level (Bonferroni adjustment).*

Statistical Analysis for H3 *- For H3, we hypothesize that the LCSDecTool intervention, compared to usual care, will result in lower rates of lung cancer screening assessed at 3 months. In this aim, the effect of the LCSDecTool intervention compared to usual care on completion of lung cancer screening will be assessed. Our analysis for H3 will be intention to treat, including all patients as randomized in the analysis using logistic regression adjusted for study site. The dependent variable will be receipt of lung cancer screening within 3 months of the index visit; the primary independent variable will be intervention group, and study site will also be included as a covariate in all analyses. Analyses will also be adjusted for characteristics not balanced by randomization as described above. Because H3 tests only one outcome, tests will be conducted at the two-sided α=0.05 level*

9.3. **Provide sample size determination and analysis (include anticipated rate of screen failures, study discontinuations, lost to follow-up, etc.)**

The sample size includes up to 200 Veterans across the 3 sites.In addition, up to 240 providers across the 3 sites with80 Providersat the CMCVAMC site,80 Providers at the West Haven CT Site, and 80 providers at the Milwaukee WI Site.

9.4. **Describe how, where and by whom the data will be analyzed**.

Power and Sample Size - We will enroll 200 persons in this pilot RCT, a sample size that is feasible to enroll and that will provide adequate power to permit testing of key study outcomes.

- *H1: We conducted our power analysis comparing the LCSDecTool to usual care with respect to the two primary outcomes, Decisional Regret and Decisional Conflict, assessed at T2 (1-month post-intervention). Assuming SD = 16.6 [95]. For the Decisional Conflict outcome, we will have 96% power to detect a medium-sized standardized effect size of 0.5, corresponding to a difference of 2.0 points between groups assuming SD = 3.9. In exploratory analyses of Africa. We will conclude that the intervention was superior if we reject the null hypothesis of no difference for either or both of these outcomes. Tests will be two-sided and will be conducted using α=0.025 to control the family-wise type I error rate at 0.05. Assuming 10% loss to follow-up at one-month*

APPROVED by CMCVAMC IRB on 03/28/2021

post-intervention, 180 patients will be available for analysis. For Decisional Regret at T2, we will have 96% power to detect a medium effect size of 0.60 corresponding to a 10-point difference between intervention and control groups, an American participant, we will have 80% power to detect a standardized effect size of 0.74 or larger in either outcome.

- *H2: Using the same approach as H1, tests will be two-sided comparisons between the intervention and control group at T2 conducted at the α=0.025 level to control the family-wise type I error rate at 0.05. With180 patients available for analysis at T2, we will have 80% power to detect a medium standardized effect size of 0.46 SD. This corresponds to absolute differences between intervention and control arms of approximately 5.5 in STAI assuming SD = 12.0 and 1.6 in lung cancer worry assuming SD = 3.5. In the African American subgroup, we will have 80% power to detect a standardized effect size of 0.74 (medium).*

Section 10: References - Bibliography of cited literature

1. Brown, D.W., *Smoking prevalence among US veterans.* J Gen Intern Med, 2010. 25(2): p. 147-9.
2. Aberle, D.R., et al., *Reduced lung-cancer mortality with low-dose computed tomographic screening.* N Engl J Med, 2011. 365(5): p. 395-409.
3. Bach, P.B., et al., *Benefits and harms of CT screening for lung cancer: a systematic review.* JAMA, 2012. 307(22): p. 2418-29.
4. Force, U.S.P.S.T., *Lung cancer screening: recommendation statement.* Ann Intern Med, 2004. 140(9): p. 738-9.
5. Wender, R., et al., *American Cancer Society lung cancer screening guidelines.* CA Cancer J Clin, 2013. 63(2): p. 107-17.
6. Mazzone, P., et al., *Components Necessary for High Quality Lung Cancer Screening: American College of Chest Physicians and American Thoracic Society Policy Statement.* Chest, 2014.
7. Pinsky, P.F., et al., *The National Lung Screening Trial: results stratified by demographics, smoking history, and lung cancer histology.* Cancer, 2013. 119(22): p. 3976-83.
8. Kovalchik, S.A., et al., *Targeting of low-dose CT screening according to the risk of lung-cancer death.* N Engl J Med, 2013. 369(3): p. 245-54.
9. Black, W.C., et al., *Cost-effectiveness of CT screening in the National Lung Screening Trial.* N Engl J Med, 2014. 371(19): p. 1793-802.
10. *Proposed Decision Memo for Screening for Lung Cancer with Low Dose Computed Tomography (LDCT) (CAG-00439N)*.
11. Tammemagi, M.C., et al., *Impact of lung cancer screening results on smoking cessation.* J Natl Cancer Inst, 2014. 106(6): p. dju084.
12. Gareen, I.F., et al., *Impact of lung cancer screening results on participant health-related quality of life and state anxiety in the National Lung Screening Trial.* Cancer, 2014. 120(21): p. 3401-9.
13. Moyer, V.A. and U.S.P.S.T. Force, *Screening for lung cancer: U.S. Preventive Services Task Force recommendation statement.* Ann Intern Med, 2014. 160(5): p. 330-8.
14. Stacey, D., et al., *Decision aids for people facing health treatment or screening decisions.* Cochrane Database Syst Rev, 2011(10): p. CD001431.
15. Frosch, D.L., F. Legare, and C.M. Mangione, *Using decision aids in community-based primary care: a theory-driven evaluation with ethnically diverse patients.* Patient Educ Couns, 2008. 73(3): p. 490-6.
16. Frosch, D.L., et al., *Shared decision-making in the United States: policy and implementation activity on multiple fronts.* Z Evid Fortbild Qual Gesundhwes, 2011. 105(4): p. 305-12.
17. Frosch, D.L., K.J. Singer, and S. Timmermans, *Conducting implementation research in community based primary care: a qualitative study on integrating patient decision support interventions for cancer screening into routine practice.* Health Expect, 2011. 14 Suppl 1: p. 73-84.
18. Marshall, D., et al., *Conjoint Analysis Applications in Health - How are Studies being Designed and Reported?: An Update on Current Practice in the Published Literature between 2005 and 2008.* Patient, 2010. 3(4): p. 249-56.
19. Bridges, J.F., et al., *Conjoint analysis applications in health--a checklist: a report of the ISPOR Good Research Practices for Conjoint Analysis Task Force.* Value Health, 2011. 14(4): p. 403-13.
20. Reed Johnson, F., et al., *Constructing experimental designs for discrete-choice experiments: report of the ISPOR Conjoint Analysis Experimental Design Good Research Practices Task Force.* Value in Health, 2013. 16(1): p. 3-13.

APPROVED by CMCVAMC IRB on 03/28/2021

21. Fraenkel, L., et al., *Patients' Preferences for Treatment of Hepatitis C.* Medical Decision-making, 2010. 30(1): p. 45-57.
22. Wortley, S., et al., *Assessing stated preferences for colorectal cancer screening: a critical systematic review of discrete choice experiments.* Patient, 2014. 7(3): p. 271-82.
23. Pignone, M.P., et al., *Conjoint analysis versus rating and ranking for values elicitation and clarification in colorectal cancer screening.* J Gen Intern Med, 2012. 27(1): p. 45-50.
24. O'Connor, A.M., et al., *The effects of an 'explicit' values clarification exercise in a woman's decision aid regarding postmenopausal hormone therapy.* Health Expect, 1999. 2(1): p. 21-32.
25. Griffith, J.M., et al., *Randomized trial of presenting absolute v. relative risk reduction in the elicitation of patient values for heart disease prevention with conjoint analysis.* Med Decis Making, 2009. 29(2): p. 167-74.
26. Imaeda, A., D. Bender, and L. Fraenkel, *What is most important to patients when deciding about colorectal screening?* J Gen Intern Med, 2010. 25(7): p. 688-93.
27. Benning, T.M., et al., *The effect of presenting information about invasive follow-up testing on individuals' noninvasive colorectal cancer screening participation decision: results from a discrete choice experiment.* Value Health, 2014. 17(5): p. 578-87.
28. Prevention, C.f.D.C.a. *Cigarette Smoking in the United States: Current Cigarette Smoning Among U.S. Adults Aged 18 Years and Older*. [cited November 22, 2014; Available from: http://www.cdc.gov/tobacco/campaign/tips/resources/data/cigarette-smoking-in-united-states.html.
29. Stiggelbout, A.M., et al., *Shared decision-making: really putting patients at the centre of healthcare.* BMJ, 2012. 344: p. e256.
30. Elwyn, G., et al., *Shared decision-making and motivational interviewing: achieving patient-centered care across the spectrum of health care problems.* Ann Fam Med, 2014. 12(3): p. 270-5.
31. Baron, J., *Why expected utility theory is normative, but not prescriptive.* Med Decis Making, 1996. 16(1): p. 7-9; discussion 14.
32. Hellinger, F.J., *Expected utility theory and risky choices with health outcomes.* Med Care, 1989. 27(3):p. 273-9.
33. Nease, R.F., Jr., *Do violations of the axioms of expected utility theory threaten decision analysis?* Med Decis Making, 1996. 16(4): p. 399-403.
34. Wu, G., *The strengths and limitations of expected utility theory.* Med Decis Making, 1996. 16(1): p. 9-10; discussion 14.
35. Ferreira, M.B., et al., *Automatic and controlled components of judgment and decision-making.* J Pers Soc Psychol, 2006. 91(5): p. 797-813.
36. Trumbo, C.W., *Heuristic-systematic information processing and risk judgment.* Risk Anal, 1999. 19(3): p. 391-400.
37. Tversky, A. and D. Kahneman, *Judgment under Uncertainty: Heuristics and Biases.* Science, 1974. 185(4157): p. 1124-31.
38. Inbar, Y., J. Cone, and T. Gilovich, *People's intuitions about intuitive insight and intuitive choice.* J Pers Soc Psychol, 2010. 99(2): p. 232-47.
39. Dohmen T, F.A., Huffman D, Sunde W, *Interpreting Time Horizon Effects in Inter-Temporal Choic*. 2012, The Institute for the Study of Labor (IZA) Bonn, Germany.
40. Bandyopadhyay, D., V.S. Pammi, and N. Srinivasan, *Role of affect in decision-making.* Progress in Brain Research, 2013. 202: p. 37-53.
41. Fraenkel, L., et al., *A pilot randomized controlled trial of a decision support tool to improve the quality of communication and decision-making in individuals with atrial fibrillation.* J Am Geriatr Soc, 2012. 60(8): p. 1434-41.
42. Chien, C.H., et al., *Changes in decisional conflict and decisional regret in patients with localised prostate cancer.* J Clin Nurs, 2014. 23(13-14): p. 1959-69.
43. Center, E.-b.S.P.E., *The Effects of Shared Decision-making on Cancer Screening-A Systematic REview*. 2014, Department of Veterans Affairs Health Services Research & Development Series.
44. Stacey, D., et al., *Decision aids for people facing health treatment or screening decisions.* The Cochrane database of systematic reviews, 2014. 1: p. CD001431.
45. Kitson, A.L., et al., *Evaluating the successful implementation of evidence into practice using the PARiHS framework: theoretical and practical challenges.* Implement Sci, 2008. 3: p. 1.

APPROVED by CMCVAMC IRB on 03/28/2021

46. Rycroft-Malone, J., *The PARIHS framework--a framework for guiding the implementation of evidence based practice.* J Nurs Care Qual, 2004. 19(4): p. 297-304.
47. Rycroft-Malone, J., *Evidence-informed practice: from individual to context.* J Nurs Manag, 2008. 16(4):p. 404-8.
48. Holden, R.J. and B.T. Karsh, *The technology acceptance model: its past and its future in health care.* J Biomed Inform, 2010. 43(1): p. 159-72.
49. Schapira, M.M. and J. VanRuiswyk, *The effect of an illustrated pamphlet decision-aid on the use of prostate cancer screening tests.* J Fam Pract, 2000. 49(5): p. 418-24.
50. Schapira, M.M., et al., *Decision-making at menopause: a randomized controlled trial of a computer based hormone therapy decision-aid.* Patient Educ Couns, 2007. 67(1-2): p. 100-7.
51. Schapira, M.M., et al., *The Numeracy Understanding in Medicine Instrument: : A Measure of Health Numeracy Developed Using Item Response Theory.* Med Decis Making, 2012.
52. Neuner, J.M. and M.M. Schapira, *The importance of physicians' risk perception in osteoporosis treatment decision-making.* Journal of Clinical Densitometry, 2012. 15(1): p. 49-54.
53. Schapira, M.M., et al., *Public engagement with scientific evidence in health: A qualitative study among primary-care patients in an urban population.* Public Underst Sci, 2014.
54. Fraenkel, L., *Incorporating patients' preferences into medical decision-making.* Med Care Res Rev, 2013. 70(1 Suppl): p. 80S-93S.
55. Owsley, C., et al., *Eye Care Quality and Accessibility Improvement in the Community (EQUALITY) for adults at risk for glaucoma: study rationale and design.* Int J Equity Health, 2015. 14(1): p. 135.
56. Chhatre, S., et al., *Understanding the Racial and Ethnic Differences in Cost and Mortality Among Advanced Stage Prostate Cancer Patients (STROBE).* Medicine (Baltimore), 2015. 94(32): p. e1353.
57. Epstein, A.J. and J.D. Ketcham, *Information technology and agency in physicians' prescribing decisions.* Rand Journal of Economics, 2014. 45(2): p. 422-448.
58. Jubelt, L.E., et al., *A Qualitative Evaluation of Patient-Perceived Benefits and Barriers to Participation in a Telephone Care Management Program.* Am J Health Promot, 2015. 30(2): p. 117-9.
59. Asan, O., E. Chiou, and E. Montague, *Quantitative ethnographic study of physician workflow and interactions with electronic health record systems.* Int J Ind Ergon, 2015. 49: p. 124-130.
60. Asan, O., J. Xu, and E. Montague, *Dynamic Comparison of Physicians' Interaction Style with Electronic Health Records in Primary Care Settings.* J Gen Pract (Los Angel), 2013. 2.
61. Asan, O., et al., *How physician electronic health record screen sharing affects patient and doctor nonverbal communication in primary care.* Patient Educ Couns, 2015. 98(3): p. 310-6.
62. Hubbard, R.A., et al., *Cumulative probability of false-positive recall or biopsy recommendation after 10 years of screening mammography: a cohort study.* Ann Intern Med, 2011. 155(8): p. 481-92.
63. Hubbard, R.A., et al., *The cumulative risk of false-positive fecal occult blood test after 10 years of colorectal cancer screening.* Cancer Epidemiol Biomarkers Prev, 2013. 22(9): p. 1612-9.
64. Hubbard, R., J. Chubak, and C. Rutter, *Estimating screening test utilization using electronic health records data.* EGEMS (Wash DC), 2014. 2(1): p. 14.
65. Orem, U., *The CBC System for Choice-Based Conjoint Analysis. Version 8. Sawtooth Software Technical Paper.* 2013.
66. Series., S.S.T.P., *The CBC/HB System for Hierarchical Bayes Estimation Version 5.0 Sawtooth Software Technical Paper*. Copyright 2000-2009: Sequim, WA.
67. Affairs, U.S.D.o.V. *National Center for Veterans Analysis and Statistics*. 2014; Available from: http://www.va.gov/vetdata/.
68. Lerz KA, A.K., Taylor C, ROse MG, Federman D, Kravetz JD, Ruser C, Schwartz AR, Beck J, Rosa M, Cain HC., *Low dose-screening CT in a Veteran cohort detects more nodules and more cancers than in other studied populations*, in *American Thoracic Society*. 2014: San Diego, CA.
69. McGinnis, K.A., et al., *Validating smoking data from the Veteran's Affairs Health Factors dataset, an electronic data source.* Nicotine Tob Res, 2011. 13(12): p. 1233-9.
70. Corbin J, S.A., *Basics of Qualitative Research*. Third ed. 2008, Thousand Oaks, California: Sage Publications.
71. Fowler JF, M.T., *Standardized Survey Interviewing*. Applied Social Research Methods. Vol. 18. 1990, Newbery Park, California: Sage.
72. *Nvivo qualitative data analysis software*. 2012, QSR International Pty Ltd.
73. Holmes-Rovner, M., *International Patient Decision Aid Standards (IPDAS): beyond decision aids to*

APPROVED by CMCVAMC IRB on 03/28/2021

usual design of patient education materials. Health Expect, 2007. 10(2): p. 103-7.
74. Miller, K.M., et al., *Promoting decision aid use in primary care using a staff member for delivery.* Patient Educ Couns, 2012. 86(2): p. 189-94.
75. Schroy, P.C., 3rd, S. Mylvaganam, and P. Davidson, *Provider perspectives on the utility of a colorectal cancer screening decision aid for facilitating shared decision-making.* Health Expect, 2014. 17(1): p. 27-35.
76. Stacey, D., et al., *Decision aids for people facing health treatment or screening decisions.* Cochrane Database Syst Rev, 2014. 1: p. CD001431.
77. Lipkus, I.M., *Numeric, verbal, and visual formats of conveying health risks: suggested best practices and future recommendations.* Med Decis Making, 2007. 27(5): p. 696-713.
78. Zipkin, D.A., et al., *Evidence-based risk communication: a systematic review.* Ann Intern Med, 2014. 161(4): p. 270-80.
79. Cohen SG, S.A., *Sawtooth Software Research Paper Series: Maximum Difference Scaling: Improved Measures of Importance and Preference for Segmentation*. 2003: Sequim, WA.
80. *The MaxDiff System Technical Paper*. 2013, Sawtooth Software, Inc: Orem, Utah.
81. Doll, W.E. and C.S. Clay, *Seismic Imaging of the Puritan Batholith, Wisconsin, Using Split Array Cross-Correlator Processing.* Journal of Geophysical Research-Solid Earth and Planets, 1988. 93(B7): p.8023-&.
82. Doll, W.J., et al., *The meaning and measurement of user satisfaction: A multigroup invariance analysis of the end-user computing satisfaction instrument.* Journal of Management Information Systems, 2004. 21(1): p. 227-262.
83. MacGregor, K., et al., *Behavior-change action plans in primary care: a feasibility study of clinicians.* J Am Board Fam Med, 2006. 19(3): p. 215-23.
84. Brandes, K., et al., *The characteristics and effectiveness of Question Prompt List interventions in oncology: a systematic review of the literature.* Psychooncology, 2015. 24(3): p. 245-52.
85. Byrne, M.M., J. Weissfeld, and M.S. Roberts, *Anxiety, fear of cancer, and perceived risk of cancer following lung cancer screening.* Med Decis Making, 2008. 28(6): p. 917-25.
86. van den Bergh, K.A., et al., *Informed decision-making does not affect health-related quality of life in lung cancer screening (NELSON trial).* Eur J Cancer, 2010. 46(18): p. 3300-6.
87. Slatore, C.G., et al., *Patient-centered outcomes among lung cancer screening recipients with computed tomography: a systematic review.* J Thorac Oncol, 2014. 9(7): p. 927-34.
88. Spiegel, T.N., et al., *Psychological impact of recall on women with BRCA mutations undergoing MRI surveillance.* Breast, 2011. 20(5): p. 424-30.
89. Davis, T.C., et al., *Rapid estimate of adult literacy in medicine: a shortened screening instrument.* Fam Med, 1993. 25(6): p. 391-5.
90. Schapira, M.M., et al., *Development and validation of the numeracy understanding in medicine instrument short form.* J Health Commun, 2014. 19 Suppl 2: p. 240-53.
91. Watson, D., L.A. Clark, and A. Tellegen, *Development and validation of brief measures of positive and negative affect: the PANAS scales.* J Pers Soc Psychol, 1988. 54(6): p. 1063-70.
92. Dolan, J.G. and S. Frisina, *Randomized controlled trial of a patient decision aid for colorectal cancer screening.* Med Decis Making, 2002. 22(2): p. 125-39.
93. O'Connor, A.M., *Validation of a decisional conflict scale.* Med Decis Making, 1995. 15(1): p. 25-30.
94. Watts, K.J., et al., *Online prostate cancer screening decision aid for at-risk men: a randomized trial.* Health Psychol, 2014. 33(9): p. 986-97.
95. Brehaut, J.C., et al., *Validation of a decision regret scale.* Med Decis Making, 2003. 23(4): p. 281-92.
96. Champion, V., C.S. Skinner, and U. Menon, *Development of a self-efficacy scale for mammography.* Res Nurs Health, 2005. 28(4): p. 329-36.
97. Biener, L. and D.B. Abrams, *The Contemplation Ladder: validation of a measure of readiness to consider smoking cessation.* Health Psychol, 1991. 10(5): p. 360-5.
98. Perzynski, A., *Health risk assessment software for everyone: Testing the association of numeracy with engagement, perceived usefulness, and satisfaction*, in *Socity for Medical Decision-making*. 2014: Miami, FL.
99. Doll, W.J. and G. Torkzadeh, *The Measurement of End-User Computing Satisfaction.* Mis Quarterly, 1988. 12(2): p. 259-274.

APPROVED by CMCVAMC IRB on 03/28/2021

100. Perzynski, A., *Health risk assessment software for everyone: Testing the association of numeracy with engagement, perceived usefulness, and satisfaction*, in *Society for Medical Decision-making*. 2014: Miami, FL.
101. Elwyn, G., et al., *Developing CollaboRATE: a fast and frugal patient-reported measure of shared decision-making in clinical encounters.* Patient Educ Couns, 2013. 93(1): p. 102-7.
102. Miller Jr, D.P., et al., *Effectiveness of a web-based colorectal cancer screening patient decision aid: A randomized controlled trial in a mixed-literacy population.* American Journal of Preventive Medicine, 2011. 40(6): p. 608-615.

APPROVED by CMCVAMC IRB on 03/28/2021
