# Supplementary material for: Lung Cancer Screening Decision Aid Designed for a Primary Care Setting: A Randomized Clinical Trial
Source: JAMA Netw Open. 2023 Aug 30;6(8):e2330452. doi: 10.1001/jamanetworkopen.2023.30452 (PMC10469267; doi:10.1001/jamanetworkopen.2023.30452)
Supplement: Supplement 2. — eMethods. Study Interventions eFigure. Quality of Decision-Making Outcomes for Knowledge and Decisional Conflict eTable. Analysis of Decision Quality Outcomes Among African American or Black Participants [file jamanetwopen-e2330452-s002.pdf]

Supplementary Online Content

Schapira MM, Hubbard RA, Whittle J, et al. Lung cancer screening decision aid designed for a primary care setting: a randomized clinical trial. *JAMA Netw Open*. 2023;6(8):e2330452. doi:10.1001/jamanetworkopen.2023.30452

eMethods. Study Interventions

eFigure. Quality of Decision-Making Outcomes for Knowledge and Decisional Conflict

eTable. Analysis of Decision Quality Outcomes Among Participants Identifying as African American or Black

This supplementary material has been provided by the authors to give readers additional information about their work.

© 2023 Schapira MM et al. *JAMA Network Open*.

eMethods. Study Interventions

The Lung Cancer Screening Decision Tool (LCSDecTool) and the control intervention used in the study can be accessed at the following links.
Lung Cancer Screening Decision Tool: http://va-lung.punkave.net/ Enter 0001 for the LCSDecTool.
Control Intervention: http://va-lung.punkave.net/ Enter 0002 for the Control Intervention.

© 2023 Schapira MM et al. *JAMA Network Open*.

eFigure. Quality of Decision-Making Outcomes for Knowledge and Decisional Conflict

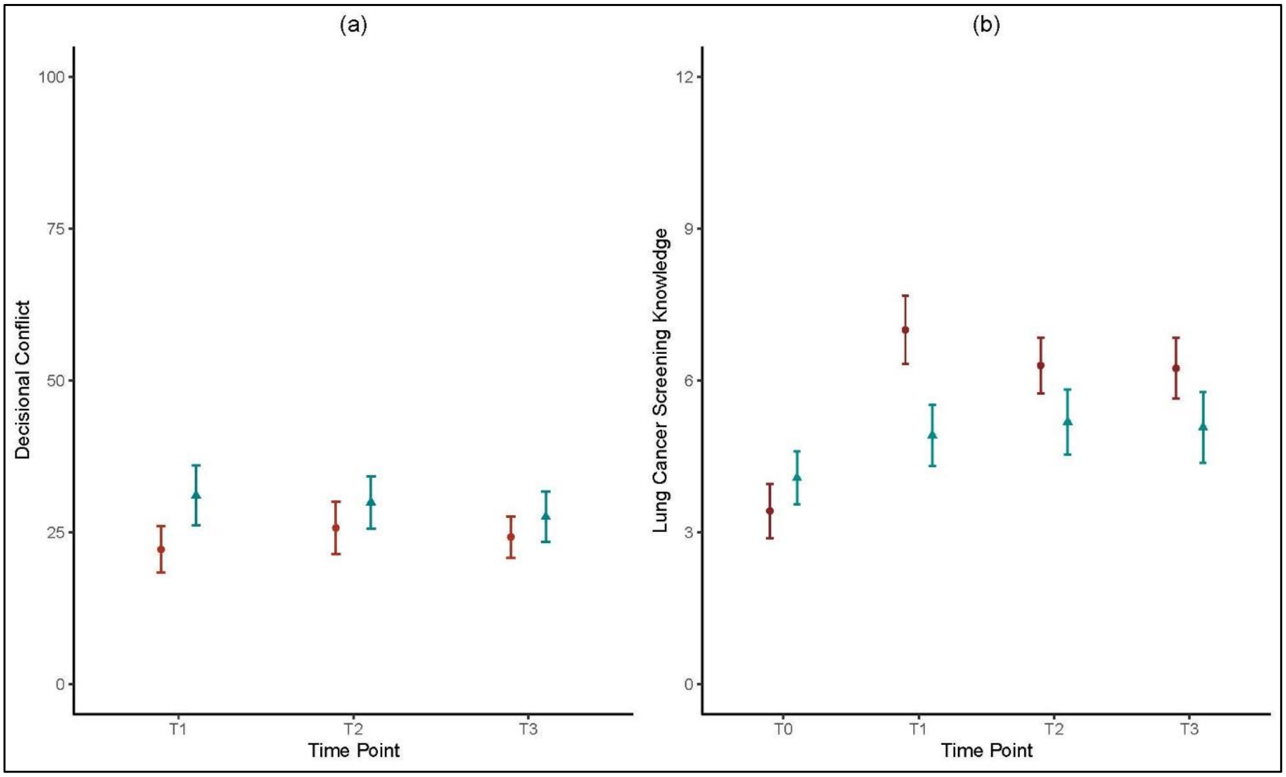

Footnote: A demonstrates the difference between the Decisional Conflict Score in the LCSDecTool Intervention Group (Red) and the Control group (Blue) at the T1 (immediate post-Intervention), T2 (1 month post Intervention) and T3 (3 months post-intervention time points. The Y axis for Figure A is the Decisional Conflict Scale with a range from 0 to 100. Figure B demonstrates Lung Cancer Screening Knowledge on a scale from 0 (Low) to 12 (High). A time T0 (Pre-intervention) there is no difference between groups, LCS Knowledge is greater in the LCSDecTool vs. Control intervention at T1 and remains greater at time points T2 and T3

© 2023 Schapira MM et al. *JAMA Network Open*.

eTable. Analysis of Decision Quality Outcomes Among Participants Identifying as African American or Black

	Control (95% CI)	Intervention (95% CI)	Difference (95% CI)	p-value
**Decisional conflict**	25.9 (19.5, 32.3)	25.3 (20.5, 30.0)	-1.0 (-9.0, 7.1)	0.82
**Decisional regret**	32.7 (29.0, 36.3)	32.9 (29.5, 36.2)	-0.5 (-5.5, 4.4)	0.83
**STAI**	36.0 (29.1, 43.0)	36.5 (32.2, 40.8)	1.3 (-5.1, 7.6)	0.69

T Unadjusted means and differences in means between control and intervention arms for usability outcomes. P-value based on t-test. Means and differences in means between control and intervention arms for primary and secondary outcomes at T2 in African-American/Black sub-group. Within-arm means and confidence intervals are unadjusted. Differences in means and their confidence intervals as well as p-values are based on linear mixed effects model adjusted for baseline value and site and including random subject intercepts.

© 2023 Schapira MM et al. *JAMA Network Open*.
