# Supplementary material for: Lung Cancer Screening Decision Aid Designed for a Primary Care Setting: A Randomized Clinical Trial
Source: JAMA Netw Open. 2023 Aug 30;6(8):e2330452. doi: 10.1001/jamanetworkopen.2023.30452 (PMC10469267; doi:10.1001/jamanetworkopen.2023.30452)
Supplement: Supplement 3. — Data Sharing Statement [file jamanetwopen-e2330452-s003.pdf]

Data Sharing Statement

Schapira. Lung Cancer Screening Decision Aid Designed for a Primary Care Setting. *JAMA Netw Open*. Published August 28, 2023. doi:10.1001/jamanetworkopen.2023.30452

Data
Data available: Yes
Data types: Deidentified participant data
How to access data: Data will be available upon request to Marilyn Schapira at the email: mschap@pennmedicine.upenn.edu
When available: With publication

Supporting Documents
Document types: Informed consent form
How to access documents: Upon request to Marilyn Schapira at email: mschap@pennmedicine.upenn.edu
When available: With publication

Additional Information
Who can access the data: Researchers whose proposed use of the data has been approved
Types of analyses: For any purpose as long as proposed use of the data is approved
Mechanisms of data availability: With investigator support after approval of proposal
Any additional restrictions: None
